# p53-regulated SESN1 and SESN2 regulate cell proliferation and cell death through control of STAT3

**DOI:** 10.1186/s12964-025-02104-3

**Published:** 2025-02-22

**Authors:** Alexander Haidurov, Andrei O. Zheltukhin, Anastasiya V. Snezhkina, George S. Krasnov, Anna V. Kudryavtseva, Andrei V. Budanov

**Affiliations:** 1 School of Biochemistry and Immunology, Trinity Biomedical Sciences Institute, Trinity College Dublin, Pearse Street, Dublin 2, Ireland; 2 Engelhardt Institute of Molecular Biology, Center for Precision Genome Editing and Genetic Technologies for Biomedicine, Moscow, Russia; 3 Shemyakin-Ovchinnikov Institute of Bioorganic Chemistry of the Russian Academy of Sciences, Moscow, Russia

**Keywords:** SESN1/2, STAT3, Lung cancer, Cell death

## Abstract

Sestrin1 and Sestrin2 (SESN1&2) are evolutionarily conserved, stress-responsive proteins that regulate cell growth and viability. The primary target of Sestrins is the mTORC1 protein kinase, an activator of anabolic processes and an autophagy inhibitor. Our previous studies showed that inactivating SESN1&2 in lung adenocarcinoma A549 cells accelerates cell proliferation and confers resistance to cell death without affecting mTORC1 activity, suggesting that SESN1&2 modulate cellular processes via mTORC1-independent mechanisms. This work describes a new mechanism through which SESN1&2 regulate cell proliferation and death by suppressing the STAT3 transcription factor. Normally activated in response to stress and inflammation, STAT3 is frequently overactivated in human cancers. This overactivation promotes the expression of pro-proliferative and anti-apoptotic genes that drive carcinogenesis. We demonstrate that SESN1&2 inactivation stimulates STAT3 by downregulating the PTPRD phosphatase, a protein responsible for STAT3 dephosphorylation. Our study demonstrates that SESN1&2 deficiency may cause STAT3 activation and facilitate carcinogenesis and drug resistance, making SESN1&2 reactivation a potential cancer treatment strategy.

**Supplementary Information:**

The online version contains supplementary material available at 10.1186/s12964-025-02104-3.

## Introduction

Lung cancer is the primary cause of cancer-related mortality around the globe, with a 5-year survival rate not exceeding 18% [1]. About 85% of lung cancers are classified as non-small cell lung cancer (NSCLC), with adenocarcinomas being the predominant tumor type [2]. The p53 protein, the product of the tumor protein *TP53* gene, plays a significant role in protecting from lung carcinogenesis, as mutations in *TP53* are found in more than 45% of human lung cancers [3]. Moreover, the inactivation of p53 in mouse models dramatically accelerates lung carcinogenesis and shortens lifespan [4]. p53 works as a direct transcriptional regulator of several hundred genes responsible for the suppression of cell proliferation, induction of cell death, regulation of metabolism, and control of reactive oxygen species [5–7]. However, the precise mechanism of suppression of carcinogenesis by p53 in the lung remains elusive and requires meticulous analysis of different p53 targets. In a search for p53 targets, we have previously characterised a new family of genes encoding stress-responsive proteins called Sestrins1-3 (SESN1-3); among them are SESN1 and SESN2 that are transcriptionally activated by p53 [7, 8]. The expression of SESN1 and SESN2 is low in p53-deficient cells [7, 8], and the expression of both genes is downregulated in human lung cancers [9, 10].

Sestrins work as multifunctional proteins responsible for suppressing reactive oxygen species (ROS), regulating mechanical target of rapamycin protein kinase complexes 1 and 2 (mTORC1/2), maintaining mitochondrial metabolism, and activating cell death [11]. mTORC1 is the primary target of Sestrins, and this kinase is responsible for the activation of cell growth and proliferation through upregulation of protein, lipid, and nucleotide biosynthesis [12, 13]. mTORC1 is activated by two types of small GTPases: RHEB and the members of the Rag family. The latter ones work as RagA/B:RagC/D heterodimers to recruit and dock mTORC1 to the lysosomal membrane, where mTORC1 is activated by RHEB, which is also anchored to the lysosomal membrane [13]. The Rag GTPases are negatively controlled by the GATOR1 protein complex that works as a GTPase-activating protein (GAP) for RagA/B. GATOR1 is inhibited by the GATOR2 protein complex, which comprises MIOS, WDR24, WDR59, SEH1L, and SEC13 subunits [13]. Sestrins suppress mTORC1 activity through direct binding of both WDR24 and SEH1L proteins within GATOR2 [14–16]. Availability of branched-chain amino acids, specifically leucine, attenuates Sestrins’ inhibition of mTORC1 by binding to their leucine-binding pocket (LBP), thus making SESN1 and SESN2 critical leucine sensors. Leucine disrupts interactions between SESN1/2 and GATOR2, leading to mTORC1 activation [17]. Our analysis of mTORC1 activity in SESN1&2 knockout (SESN1&2 KO) cells demonstrated that inactivation of SESN1&2 in lung adenocarcinoma A549 cells does not affect phosphorylation of mTORC1 targets, and the GATOR-mTORC1 pathway is not responsible for the regulation of cell death by SESN1&2 [9, 18, 19]. Therefore, Sestrins likely regulate cell signaling in lung adenocarcinoma cells through a pathway distinct from GATOR-mTORC1 axis [9].

Signal transducers and activators of transcription (STAT) is a family of transcriptional factors that play a major role in many physiological and pathophysiological processes, including stress response and inflammation. Among them, STAT3 is often found hyperactivated in lung and other cancers, contributing to carcinogenesis and drug resistance [20, 21]. STAT3 is typically located in the cytoplasm in an inactive form and is activated via phosphorylation of Tyr705 by the Janus kinase 2 (JAK2). This leads to STAT3 dimerisation and its nuclear translocation [21]. As a result, STAT3 transcriptionally activates genes encoding proteins responsible for cell proliferation (Cyclin D1 and c-Myc), inhibition of cell death (MCL1 and BCLX_L_), tissue remodelling (MMP2 and MMP9), inflammation (IL6 and IL10), and angiogenesis (VEGF and bFGF) [21, 22]. Cytokines, such as IL6, bind and activate their corresponding receptors, which dimerise and recruit JAK2. Recruitment of JAK2 molecules to their complementary receptors induces their auto-phosphorylation and activation, allowing them to phosphorylate STAT3 [21]. In parallel, STAT3 can also be activated by other tyrosine kinase family members, including EGFR, IGFR, and SRC [21]. In normal cellular conditions, STAT3 is tightly controlled by receptor and non-receptor tyrosine phosphatases, such as PTPRD and SHP1/2, respectively. STAT3 is also negatively regulated by the SOCS family members, which inhibit the activity of JAK kinases through the control of their ubiquitination and degradation, and by PIAS proteins that bind STAT3 and inhibit its transcriptional activity [21–23]. However, STAT3 is constitutively active in many cancers, supporting cell proliferation, invasiveness, and the cancer stemness phenotype [21, 23]. Hyperactivity of STAT3 in NSCLCs is associated with poor prognosis, and its persistent phosphorylation can be found as commonly as in 65% of NSCLCs [24]. STAT3 hyperactivation could be caused by many factors, including cytokine production by cancer and immune cells, activation of oncogenes, and inactivation of tumor suppressor proteins [21]. However, the precise mechanisms responsible for continuous STAT3 activation in lung cancers are poorly understood.

In a search for new Sestrins targets, we identified STAT3 as a protein whose phosphorylation is increased in SESN1&2-deficient A549 cells. Moreover, we have demonstrated that the increased STAT3 activity is responsible for accelerated cell proliferation and resistance to genotoxic drugs. Therefore, we propose that SESN1&2 may act as tumor suppressors in lung and other cancer types by suppressing aberrant STAT3 activation, protecting against uncontrolled cell proliferation, and maintaining cell death.

## Results

### Knockout of SESN1 and/or SESN2 activate STAT3

**Fig. 1:**
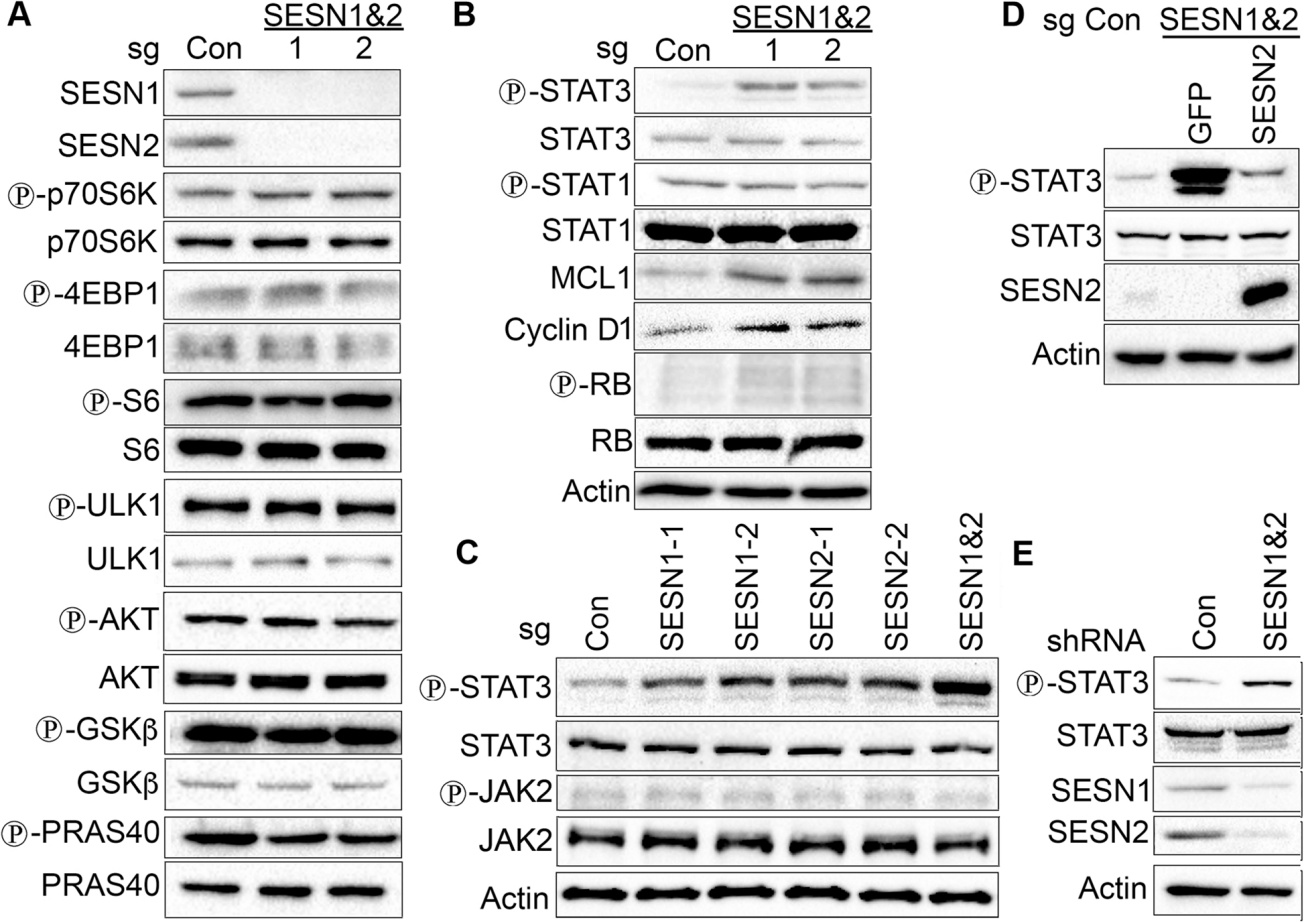
Inactivation of SESN1 and SESN2 activates STAT3 in A549 cells. **A** Immunoblot analysis of phosphorylation and expression of the components of AKT-mTOR signaling pathway in control (sgCon) and SESN1&2 KO (sgSESN1&2) cells. **B** Inactivation of SESN1&2 increases STAT3 phosphorylation, MCL1 and Cyclin D1 expression, and phosphorylation of RB. **C** Inactivation of either SESN1 (sgSESN1) or SESN2 (sgSESN2) has an intermediate effect on STAT3 phosphorylation comparatively to inactivation of both (sgSESN1&2); inactivation of SESN1, SESN2, or both does not affect JAK2 phosphorylation. **D** Ectopic SESN2 expression restores STAT3 phosphorylation in SESN1&2 KO cells to the levels observed in control cells. **E** Cells with knockdown of SESN1&2 (shRNA SESN1&2) demonstrate increased STAT3 phosphorylation levels comparatively to control cells (shRNA Con) (B-E). The results shown in A and B represent the same cellular extracts and the loading control represents both panels. Phosphorylation and expression of the indicated proteins were analysed by immunoblotting

To study the potential role of the SESN1 and SESN2 in the regulation of cell proliferation and cell death, we examined several signaling pathways that can be regulated by Sestrins in accordance with previous studies. To investigate Sestrins’ inhibitory effect on mTORC1 [25], we analysed phosphorylation of mTORC1 targets (p70S6K, S6, 4EBP1, and ULK1) in control and two distinct SESN1&2 knockout A549 cell lines [9]. We could not detect any significant difference in phosphorylation of any of these proteins, indicating that SESN1&2 do not affect mTORC1 activity in these cells in a nutrient- and growth factor- repleted cell culture medium (Fig. 1A). To measure the activity of mTORC2-AKT signaling pathway, which can be positively regulated by Sestrins, we determined the phosphorylation of AKT and its targets (GSK-β and PRAS40). However, we did not detect any notable difference in the regulation of any of these proteins. We noticed a slight decrease in the phosphorylation of the negative regulator of mTORC1, the PRAS40 protein, however, it did not affect mTORC1 activity according to phosphorylation of any mTORC1 targets (Fig. 1A). It was also reported that Sestrins might regulate AMPK, MAPK, and WNT/β-Catenin [26–28]. Considering these possibilities, we analysed the phosphorylation of AMPK, the MAPK family members (ERK, JNK and p38) and β-Catenin. We did not observe any notable difference in phosphorylation of these proteins between control and SESN1&2 KO cells (Figure S1A). Sestrins can also regulate cellular processes by influencing the degradation of SQSTM1/p62, which facilitates autophagy and supports carcinogenesis [29, 30]. However, there was no difference in the levels of p62 expression in control and SESN1&2 KO cells (Figure S1A).

The STAT3 transcription factor is often overactivated in cancers and plays an essential role in control of cell proliferation and cell death. Strikingly, we observed higher levels of STAT3 phosphorylation in SESN1&2-deficient cells compared to control cells, but SESN1&2 inactivation did not affect the phosphorylation of STAT1, another member of the STAT family (Fig. 1B). We also found that the expression of two STAT3 targets was upregulated: induced myeloid leukemia cell differentiation protein (MCL1), a suppressor of apoptotic cell death, and Cyclin D1, a cell cycle activator. Accordingly, phosphorylation of the retinoblastoma protein (RB), the major inhibitor of G1-S cell cycle transition, was also increased (Fig. 1B).

To study the impact of each Sestrin gene on the regulation of STAT3, we tested its phosphorylation in single SESN1/2 and double SESN1&2 KO cells, that were previously described [9]. Inactivation of either SESN1 or SESN2 displays intermediate levels of STAT3 phosphorylation between control and double KO cell lines (Fig. 1C), demonstrating that both genes contribute to the negative regulation of phosphorylation. We also examined the phosphorylation of upstream STAT3 kinase JAK2 but did not observe any difference in JAK2 phosphorylation between control and SESN1- or SESN2- deficient cell lines, indicating that STAT3 phosphorylation is controlled downstream of JAK2. To rule out the potential non-specific activity of the CRISPR constructs and confirm that SESN1&2 proteins regulate STAT3, we ectopically expressed SESN2 or both SESN1&2 proteins in SESN1&2 KO cells. We observed that the levels of STAT3 phosphorylation were restored in the SESN2 or SESN1&2 reconstitutes to the levels observed in control cells (Fig. 1D, S1B). In parallel, we also silenced the expression of both SESN1 and SESN2 in A549 cell lines by lentiviral vectors expressing corresponding shRNAs [25] and demonstrated increased STAT3 phosphorylation in SESN1&2 knockdown cells (Fig. 1E). To rule out exclusiveness of the effect of Sestrins on STAT3 in A549 cells, we also silenced SESN2 in another lung adenocarcinoma cell line, H460, and observed upregulation of STAT3 phosphorylation in these cells (Figure S1C).

### Regulation of STAT3 phosphorylation by SESN1&2 is independent of GATOR2 and requires JAK activity

**Fig. 2:**
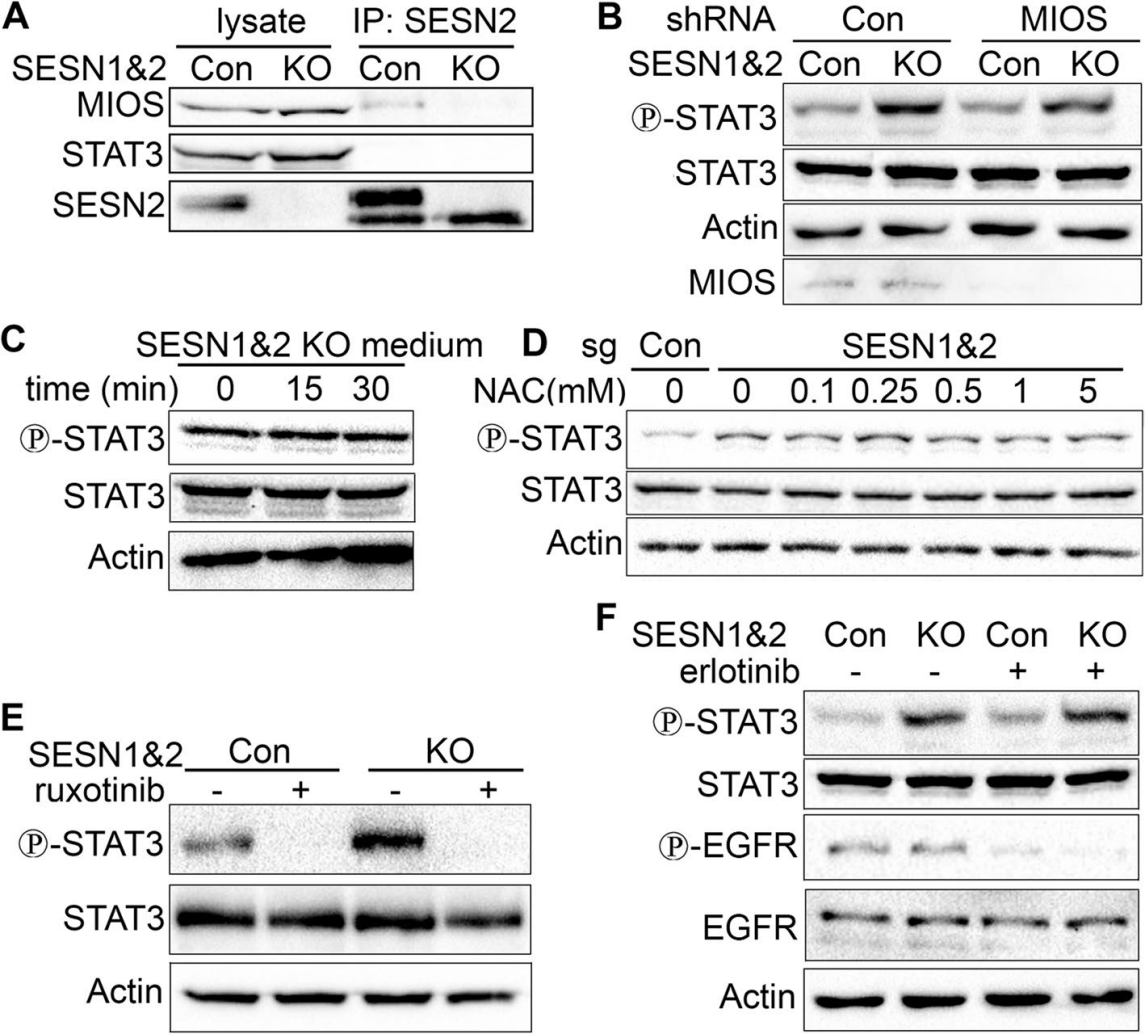
STAT3 phosphorylation in SESN1&2 KO A549 cells is JAK-dependent. **A** SESN2 does not interact with STAT3. SESN2 and SESN2-interacting proteins were immunoprecipitated (IP) with anti-SESN2 antibody. **B** Regulation of STAT3 by SESN1&2 does not involve GATOR2. MIOS was silenced by shRNA (shRNA MIOS) in control and SESN1&2 KO cells. **C** SESN1&2 does not regulate STAT3 phosphorylation through secreted factors. Control A549 cells (sgCon) were incubated with media from SESN1&2 KO cells (sgSESN1&2) for the indicated time intervals. **D** Activation of STAT3 phosphorylation in SESN1&2 KO cells does not depend on antioxidant activity of Sestrins. SESN1&2 KO cells were treated with different concentrations of a ROS scavenger NAC for 3 h. **E**–**F** Treatment with JAK inhibitor ruxolitib (0.5 µM) (E), but not EGFR inhibitor erlotinib (500 nM) (F), abrogates STAT3 phosphorylation in control and SESN1&2 KO cells. Phosphorylation and expression of the indicated proteins were analysed by immunoblotting

In an attempt to determine the role of SESN1&2 in the control of STAT3 phosphorylation, we proposed that Sestrins may directly interact with the STAT3 protein, suppressing its phosphorylation by upstream kinases or activating dephosphorylation by phosphatases. To validate this assumption, we performed immunoprecipitation analysis using an approach we previously used to analyse the interactions between SESN2 and the GATOR2 protein complex, the major Sestrin interactor [14, 31]. SESN2 protein was immunoprecipitated with anti-SESN2 antibodies and the presence of STAT3 and GATOR2 component MIOS were analysed by immunoblot. We did not observe any co-precipitation of STAT3 with SESN2, while MIOS was present in the complex (Fig. 2A, S2). We also determined whether GATOR2 mediates the suppression of STAT3 phosphorylation by SESN1&2. To examine this possibility, we silenced MIOS in the control and SESN1&2 KO cells and examined STAT3 phosphorylation by immunoblot. MIOS silencing did not reveal any observable effects on STAT3 phosphorylation in both control and SESN1&2 KO cells (Fig. 2B). SESN2 is known for its suppression of inflammation and antioxidant activity [29, 32], so we determined if STAT3 phosphorylation in SESN1&2 KO cells may have been mediated by the secretion of growth factors or cytokines into the medium or elevation of ROS. To define whether SESN1&2 regulate STAT3 via an indirect mechanism mediated by elevated production of secreted factors, we replaced the medium of the control cells with the medium collected from the SESN1&2 KO cells. We did not observe any increase in STAT3 phosphorylation in control cells incubated with medium from SESN1&2 KO cells, ruling out the impact of secreted factors in the regulation of STAT3 phosphorylation by Sestrins (Fig. 2C). To determine the potential role of ROS in STAT3 phosphorylation in SESN1&2 KO cells, we treated SESN1&2 KO cells with different concentrations of ROS scavenger N-acetyl cysteine (NAC) and analysed the levels of STAT3 phosphorylation in treated and untreated cells. The application of different concentrations of NAC did not affect STAT3 phosphorylation, indicating that the antioxidant activity of SESN1&2 does not play any remarkable role in this process (Fig. 2D).

Pursuing the goal of understanding the impact of SESN2 in better detail, we tested if the regulation of STAT3 phosphorylation by SESN1&2 requires JAK kinases, which are activated by cytokines and several growth factors through activation of their cognate receptors. To determine the importance of JAKs in STAT3 regulation in control and SESN1&2 KO cells, we treated cells with the JAK inhibitor ruxolitinib and observed that STAT3 was completely dephosphorylated in both control and SESN1&2 KO cells, indicating that JAKs are required for the phosphorylation of STAT3 in both control and SESN1&2 KO A549 cells (Fig. 2E). EGFR, the critical regulator of carcinogenesis in lung adenocarcinoma cells, may also be responsible for STAT3 phosphorylation in this context. To check if EGFR is implicated in STAT3 regulation, we treated control and SESN1&2 KO cells with the EGFR inhibitor erlotinib and analysed STAT3 phosphorylation. While erlotinib strongly inhibited EGFR phosphorylation, it did not affect STAT3 phosphorylation in control or SESN1&2 KO cells, ruling out the potential impact of EGFR in this process (Fig. 2F).

### SESN1&2 regulate the expression of genes involved in the regulation of the JAK2-STAT3 pathway

To analyse the potential impact of Sestrins in the regulation of STAT3, we evaluated expression profiles changes in Sestrin knockouts by RNA-seq. The gene expression level changes were significant and there were about 6 thousand differentially expressed genes (DEGs; FDR < 0.05), which accounted for 30–45% of all genes included in the analysis (Figure S3). For 1.3–1.7 thousand DEGs, the amplitude of expression level change was more than twofold. Within each knockout, all replicates were in good agreement with each other. There was a partial consistency between two knockouts (Spearman’s *r* = 0.22 for log2 fold changes). There were 1852 common DEGs (12%) between two knockouts (FDR < 0.05) with matching directions of expression change, and for 1060 DEGs (7%) the directions were opposite.

Next, we performed enrichment analysis for DEGs using the Gene Ontology database. For both SESN1&2 knockout variants, we revealed enrichment of downregulated genes involved in organism and tissue development (GO:0048856), including development of epithelium, skin, blood vessel, digestive tract, diaphragm, nervous system, as well as cell differentiation (GO:0030154) and other related processes. In addition, we observed enrichment of downregulated DEGs responsible for cell adhesion (GO:0007155), migration and motility, immune system processes (GO:0002376). For both knockouts, a preferential increase in the expression of genes involved in fatty acids oxidation (GO:0019395) was observed. Many processes enriched in DEGs were also identified, for which, however, there was no consistency between the knockouts.

Nevertheless, according to GO enrichment data, we did not observe significant (and consistent between knockouts) enrichment of genes directly involved in any signaling pathways, including JAK-STAT. In many respects, this is quite expected, since a change in the activity of a signaling pathway (e.g., by phosphorylation of its member) will primarily be manifested by changes in the expression of its downstream DNA targets, and more rarely – in the expression of genes that are direct participants of this pathway.

Given this fact, we performed transcription factor (TF) target enrichment analysis using ChEA3 for the revealed DEGs. Since the RNA-seq results for the two knockouts were somewhat different from each other, we first generated a ranked gene list based on the maximum (‘worst’) *p*-values among two knockouts (in the case of the opposite LogFCs, the gene was excluded) and used it for ChEA3 analysis. Across a range of databases (both ChIP-Seq and co-expression) included in ChEA3, the results were significantly different. According to the ENCODE ChIP-Seq data, we observed a strong enrichment of STAT1, STAT2 and STAT3 targets among top lists of either overexpressed or downregulated genes (Table S4,S5). Thus, 25 of top 200 upregulated and 30 of top 200 downregulated genes contain STAT3 binding site, and the enrichment p-value < 0.05 for all STAT1, STAT2, and STAT3 (according to ENCODE ChIP-Seq). However, the enrichment was not confirmed by other datasets (Remap ChIP-Seq or co-expression databases – ARCHS4, GTEx). Depending on the list size, the enrichment results also varied.

Thus, the TF target enrichment analysis suggest that the activity of the JAK-STAT signalling pathway is obviously modulated, although no unambiguous direction of JAK-STAT modulation can be stated here.

### SESN1&2 regulate the expression of genes involved in the regulation of the JAK2-STAT3 pathway

**Fig. 3:**
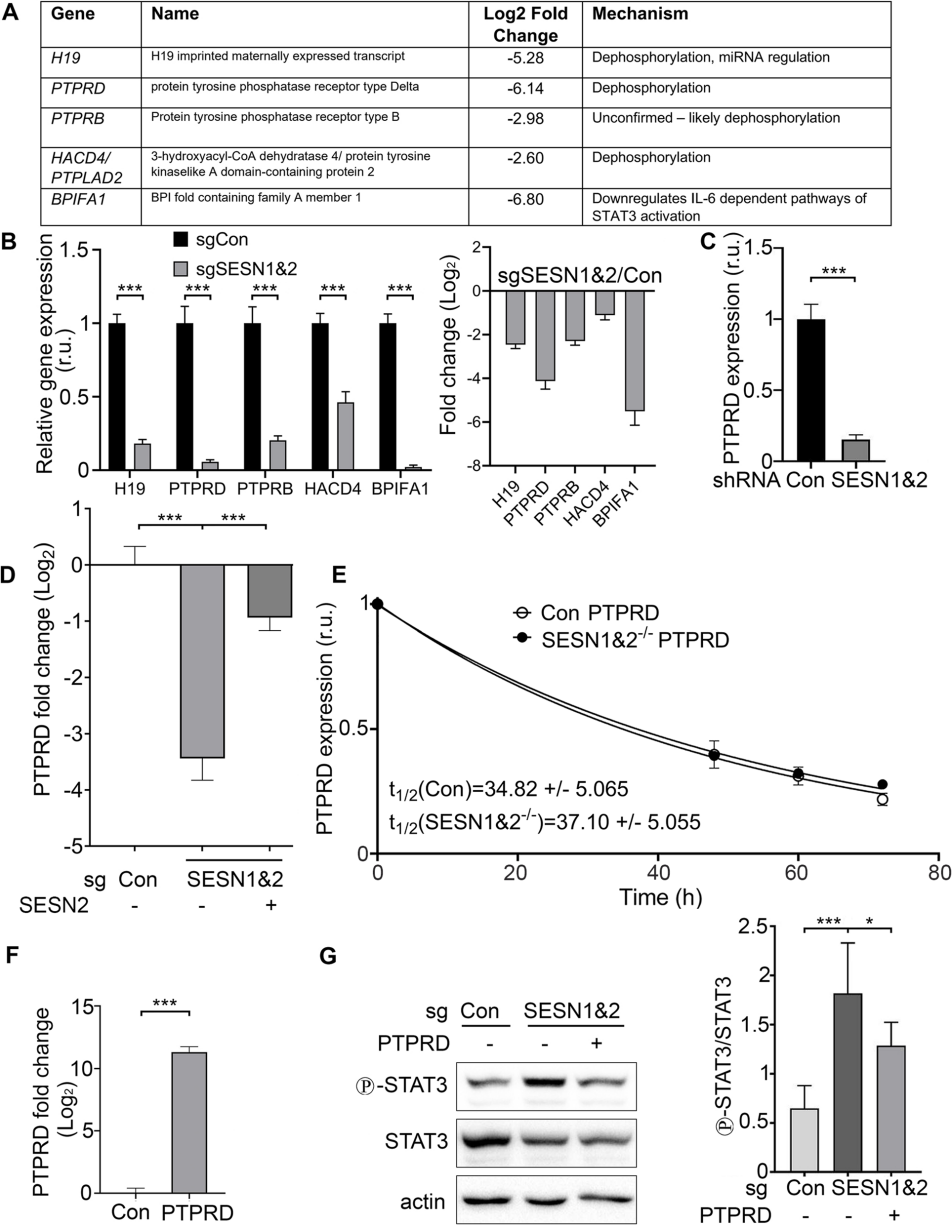
Inactivation of SESN1&2 downregulates expression of PTPRD and several other potential STAT3 regulators. **A**-**B** Potential STAT3 regulators are downregulated in SESN1&2 KO cells. **A** The results of RNA-seq analysis showing the genes whose expression is downregulated in SESN1&2 KO cells comparatively to control. Genes were identified based on FDR < 0.05. The table displays values of base twofold-change in mRNA expression in SESN1&2 KO vs control cells. **B** qPCR validation of downregulation of the aforementioned genes. The bar chart displays the mean relative gene expression ± S.D. in SESN1&2 KO vs control cells (*n* = 2). The second bar chart displays the same data as mean LogFC ± S.D. Cells were plated with the same density, cDNA was synthesized and analysed by SYBR-green-based qPCR. **C** PTPRD expression is decreased in A549 cells where SESN1&2 expression was silenced by shRNAs. PTPRD expression was analysed as in B (*n* = 2). **D** Ectopic expression of SESN2 in SESN1&2 KO cells partially restores PTPRD expression to the levels observed in control cells. The SESN2-expressing construct was delivered in a lentiviral vector, and PTPRD expression was analysed by qPCR as shown in B (*n* = 2). **E** SESN1&2 do not regulate PTPRD mRNA degradation. Control and SESN1&2 KO cells were treated with Actinomycin D (10 µg/ml) for indicated time intervals and the relative mRNA expression levels were analysed by qPCR. **F**-**G** Ectopic expression of PTPRD restores STAT3 phosphorylation in SESN1&2 KO A549 cells. **F** The SESN1&2 KO cells were infected with PTPRD-expressing lentiviral construct and analysed by qPCR (*n* = 2). **G** Ectopic PTPRD expression in SESN1&2 KO (sgSESN1&2) cells restores STAT3 phosphorylation levels similar to those observed in control cells. Gene expression quantified by qPCR was normalised to the expression of β-actin in the corresponding samples. Densitometry on immunoblots with ectopic expression of PTPRD was performed using Bio-Rad Image Lab Software. The bar chart represents STAT3’s mean phosphorylation:expression ratio ± S.D. *P* values were calculated using one-way ANOVA followed by Tukey’s post-test comparison (*n* = 6). p* ≤ 0.05; p*** ≤ 0.001

Next, we focused on five genes (*H19*, *PTPRD*, *PTPRB*, *HACD4/PTPLAD2*, and *BPIFA1*) that were previously reported to be involved in the regulation of the JAK2-STAT3 pathway (Fig. 3A). For all these genes, RNA-seq data revealed decreased expression in SESN1&2 KO cells comparatively to control cells, and this was confirmed by qPCR (Fig. 3B). While most of these genes’ precise role in regulating STAT3 is not well-defined, it was shown that tyrosine phosphatase PTPRD can dephosphorylate STAT3 in cancer cells [33]. To confirm that downregulation of PTPRD in SESN1&2 KO cells is the consequence of the inactivation of SESN1 and SESN2, we analysed PTPRD expression in A549 cells where SESN1 and SESN2 were silenced by shRNA. We found that PTPRD mRNA levels were strongly decreased in the cells with SESN1&2 knockdown (Fig. 3C). In a parallel study, we also analysed PTPRD expression in the SESN1&2 KO cells ectopically expressing SESN2. Ectopic expression of SESN2 in SESN1&2 KO cells partially restored the PTPRD expression to similar levels that were observed in control cells (Fig. 3D). mRNA expression can be regulated via control of its transcription or degradation. To discriminate between these two possibilities, we treated cells with Actinomycin D, the inhibitor of RNA synthesis, and measured RNA levels during the course of the treatment. We observed no difference in the rate of reduction of the PTPRD mRNA levels at different time intervals after the onset of Actinomycin D application, indicating that the rate of RNA degradation was comparable between control and SESN1&2 KO cells (Fig. 3E). To determine whether PTPRD mediates the negative regulation of STAT3 phosphorylation by SESN1&2, we expressed PTPRD in the SESN1&2 KO cells (Fig. 3F) and demonstrated that its ectopic expression caused a significant reduction in STAT3 phosphorylation levels in SESN1&2 KO cells (Fig. 3G).

### Inactivation of SESN1&2 provides resistance to cisplatin

**Fig. 4:**
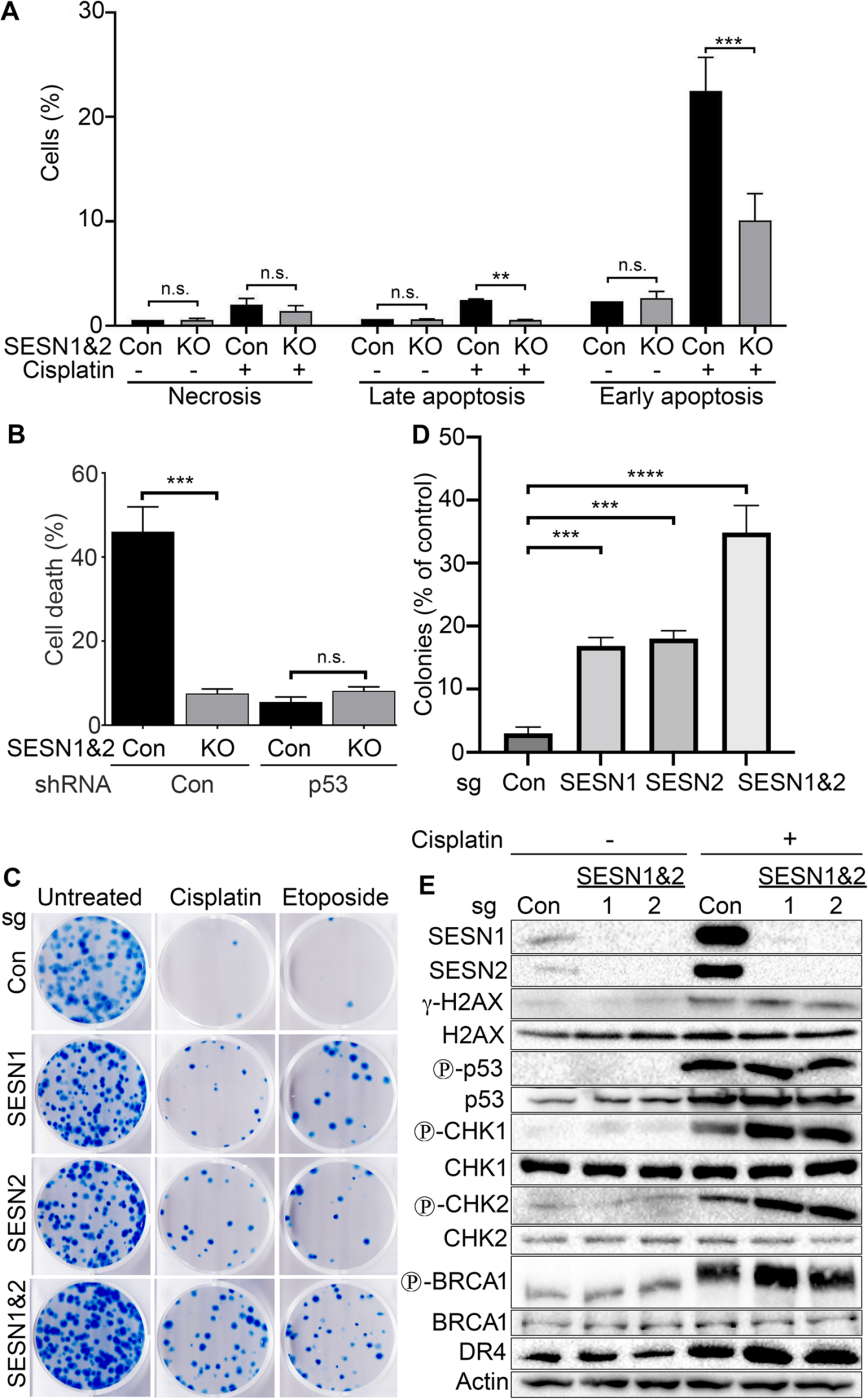
SESN1&2 are responsible for the activation of cell death in response to cisplatin. **A** SESN1&2 inactivation in A549 cells confers resistance against DNA-damage-induced apoptosis. Cells were treated with cisplatin (20 µM) for 24 h, stained by AnnexinV;PI and analysed by flow cytometry. Bar chart separates Necrosis (PI + ;AnnexinV-), Late Apoptosis (PI + ;AnnexinV +), and Early Apoptosis (PI-;AnnexinV +) to show a difference in cell death between SESN1&2 KO and control cells. The data are presented as mean ± S.D. (*n* = 4). *P* values were calculated using two-way ANOVA, followed by Tukey’s post-test comparison. **B** SESN1&2-regulated cell death is p53-dependent. Silencing of p53 by shRNA strongly inhibits cisplatin-induced cell death and eliminates the difference in cell death between the control and SESN1&2 KO cells. Control and SESN1&2 KO cells expressing either shp53 or shCon were treated with cisplatin (20 µM), stained with AnnexinV, and analysed by flow cytometry. **C** SESN1&2 KO cells form a substantially higher number of colonies than control cells in response to cisplatin (20 µM) and etoposide (20 µM). 500 cells per well were plated onto a 6-well plate and treated with the chemotherapeutic drugs for 6 h. Cell colonies were growing for two weeks, stained with methylene blue, and counted. Bar chart (**D**) represents data from cisplatin-treated colony formation experiments. The data are presented as mean ± S.D. *P* values were calculated using one-way ANOVA, followed by Tukey’s post-test comparison (*n* = 3). p** ≤ 0.01; p*** ≤ 0.001; p**** ≤ 0.0001. **E** SESN1&2 do not affect the regulation of the DDR pathway. Control (sgCon) and SESN1&2 KO (sgSESN1&2) cells were treated with cisplatin (20 µM) for 24 h and the expression and phosphorylation of the indicated proteins were analysed by immunoblotting

Our previous data indicate that SESN2 supports cell death in response to genotoxic chemotherapeutic drugs [8], and inactivation of SESN1&2 in cancer cells potentially provides resistance to cell death induced by DNA damage. SESN1&2 are direct targets of p53 and are strongly upregulated in response to genotoxic stress in a p53-dependent manner. Platinum-based drugs, such as cisplatin, are amongst the most widely used medications to treat lung cancers. To determine whether SESN1&2 are responsible for induction of cell death in response to cisplatin, we analysed cell death in cells treated with cisplatin by AnnexinV;PI staining followed by flow cytometry analysis. As shown in Fig. 4A, inactivation of SESN1&2 strongly suppresses apoptotic cell death in response to cisplatin, supporting our hypothesis that Sestrins play an important role in cell death activation in response to genotoxic stress. We tested whether SESN1&2 mediate p53’s role in triggering cell death due to DNA damage. To analyse p53’s influence, we knocked down p53 by shRNA in both control and SESN1&2 KO cells (Figure S6) [7] and compared cisplatin-induced cell death between control and p53-silenced cells. Knockdown of p53 confers resistance to cisplatin in SESN1&2-proficient cells and eliminates the disparity in cell death levels between control and SESN1&2 KO cells in response to treatment with the genotoxic drug (Fig. 4B). To investigate whether GATOR2 is involved in mediating SESN1&2-induced cell death, we determined the levels of cell death in control and SESN1&2 KO cells expressing MIOS shRNA. The data demonstrate that MIOS knockdown does not affect the levels of cell death in control and SESN1&2 KO cells, ruling out the potential impact of GATOR2 in this process (Figure S7).

To study the impact of SESN1&2 in the regulation of long-term viability and propagation of A549 cells, we performed colony formation assays with control and SESN1&2-null cells treated with cisplatin. Inactivation of either SESN1 or SESN2 strongly increases the number of colonies grown after cisplatin treatment, and double SESN1&2 KO cells produce the largest number of colonies (Fig. 4C-D). To confirm that the inactivation of SESN1&2 provides resistance to genotoxic stress and not specifically to cisplatin, we also treated cells with etoposide and observed a dramatic increase in colony formation in the cells with inactivation of SESN1 and/or SESN2 (Fig. 4C).

The mechanism of cell death regulation by Sestrins remains unknown and may involve the modulation of the activity or stability of some proteins regulating the DNA damage response (DDR). To determine whether Sestrins regulate cell death by affecting the DDR pathway, we analysed the phosphorylation and expression of key proteins of this pathway. We found that SESN1&2 are strongly upregulated by cisplatin in control cells, but neither SESN1 nor SESN2 were detected in the knockout cells (Fig. 4E). Comparing phosphorylation and expression of the proteins involved in the DDR in control and SESN1&2 KO cells, we observed a slight increase in the phosphorylation of DDR regulators: Chk1, Chk2 and BRCA1 [34]. However, we found no difference in the phosphorylation of H2A.X (a marker of DNA damage) or p53 between control and SESN1&2 KO cells. Moreover, no difference was observed between total p53 levels and expression of proapoptotic DR4 protein that is regulated in a p53-dependent manner [35] (Fig. 4E). These data indicate that SESN1&2 presumably do not play a critical role in regulating DDR and p53 activity but might be responsible for controlling cell death downstream of p53.

### Regulation of cell proliferation and cell death by SESN1&2 is mediated by STAT3

**Fig. 5:**
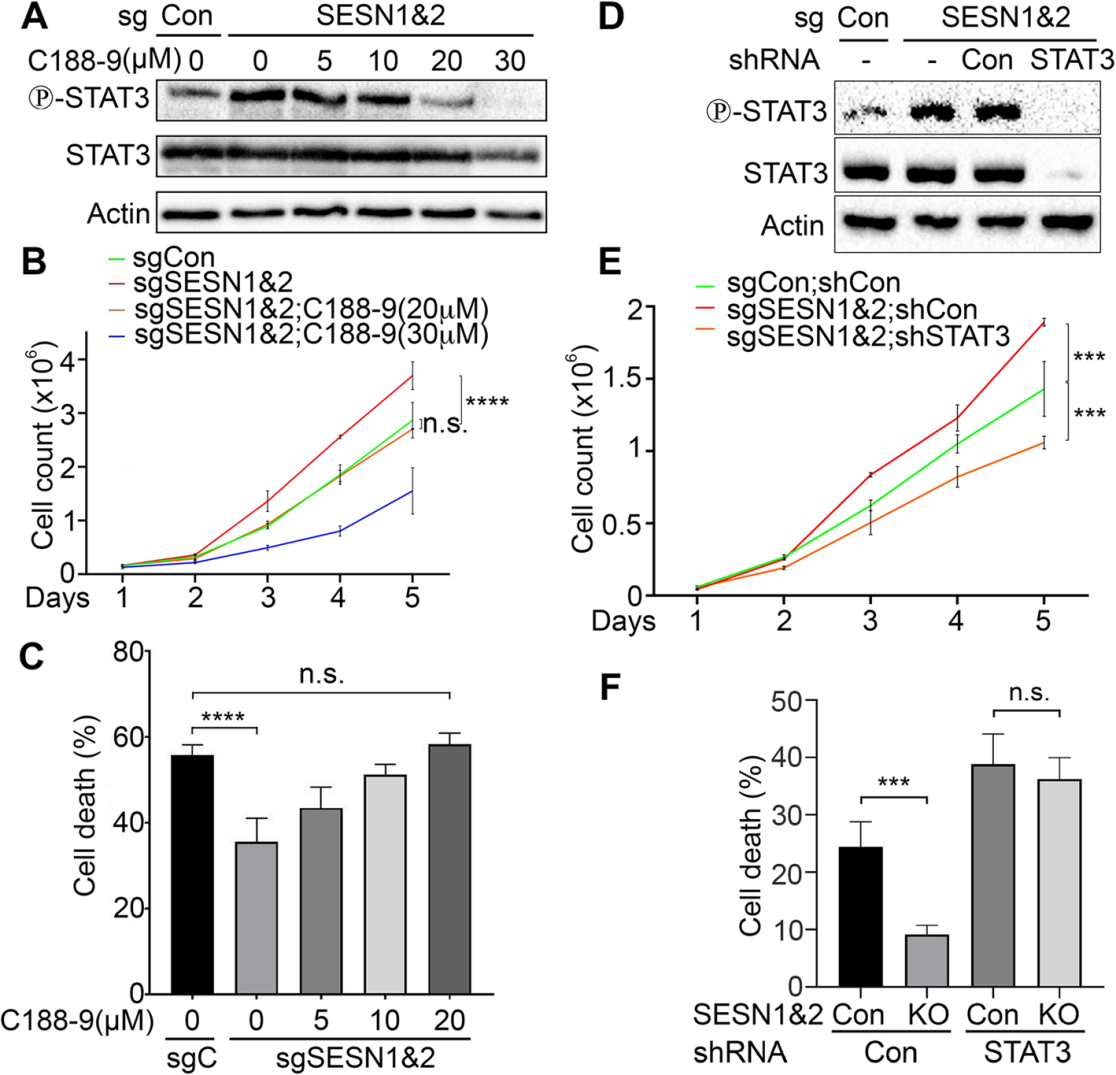
SESN1&2 regulate cell proliferation and cell death via a STAT3-dependent mechanism **A** Analysis of STAT3 phosphorylation in response to different concentrations of STAT3 inhibitor C188-9. SESN1&2 KO (sgSESN1&2) cells were treated for 24 h with corresponding concentrations of C188-9, and phosphorylation and expression of STAT3 were determined by immunoblotting. **B** Treatment of SESN1&2 KO cells with C188-9 reverses the proliferation rate to the levels observed in control cells. 50,000 cells were plated on 6-cm culture dishes and the number of cells was calculated every 24 h by haemocytometer. **C** Treatment of SESN1&2 KO cells with C188-9 reverses the apoptotic rate to the levels observed in control cells. Cells were co-treated with cisplatin (20 µM) and varying concentrations of C188-9 for 24 h. The levels of cell death were analysed by AnnexinV staining followed by flow cytometry. The data in B and C are presented as mean ± S.D. *P* values were calculated using two-way ANOVA, followed by Tukey’s post-test comparison. **D** Silencing of STAT3 by shRNA (shRNA STAT3) effectively inhibits its expression in SESN1&2 KO cells. Cells were infected with a shSTAT3-expressing lentiviral construct, selected on hygromycin, and the levels of STAT3 phosphorylation and expression were analysed by immunoblotting. **E** STAT3 silencing suppresses proliferation of SESN1&2 KO cells. Growth curve was obtained as in B. **F** STAT3 silencing attenuates SESN1&2 KO resistance to cisplatin to the levels observed in control cells. Control and STAT3-silenced cells were treated with cisplatin (20 µM) for 24 h, and the levels of apoptosis were analysed as in C. The data in E and F are presented as mean ± S.D. *P* values were calculated using one-way and two-way ANOVA, followed by Tukey’s post-test comparison. p*** ≤ 0.001; p**** ≤ 0.0001

STAT3 plays a crucial role in cell proliferation and suppression of cell death in many tumor cells. SESN1&2 KO cells demonstrate higher proliferation rates and greater resistance to cell death than control cells. To examine the role of STAT3 in the regulation of cell proliferation and cell death, we analysed STAT3 phosphorylation in the SESN1&2 KO cells treated with different concentrations of STAT3 inhibitor C188-9. This small molecule selectively targets the pY-peptide binding site within the STAT3 SH2 domain, blocking its phosphorylation [36]. Treatment with 20 µM of C188-9 decreased STAT3 phosphorylation to the levels observed in control cells, and incubation with 30 µM of C188-9 almost eliminated STAT3 phosphorylation (Fig. 5A). We determined proliferation rates in control cells and SESN1&2 KO cells after treating them with different concentrations of C188-9. As previously shown [9], SESN1&2 KO cells grow faster than control cells, and treatment with 20 µM of C188-9 slows down proliferation of SESN1&2 KO cells to the rate observed in the control cells. Moreover, the treatment with 30 µM of C188-9 decreases the cell proliferation rate even further (Fig. 5B). To study whether the activation of STAT3 in SESN1&2 KO cells is responsible for controlling cell death, we treated cells with cisplatin in the presence of different concentrations of C188-9. We demonstrated that treatment of SESN1&2 KO cells with 20 µM of C188-9 induced cell death to similar levels observed in the control cells (Fig. 5C). Similar effects on cell death were observed when cells were treated with JAK inhibitor ruxotinib (Figure S8) but not an inhibitor of non-receptor tyrosine kinases dasatinib (Figure S9). To rule out any non-specific activity of the STAT3 inhibitor, we silenced STAT3 with shRNA and generated an almost complete knockdown of STAT3 in the control and SESN1&2 KO cells (Fig. 5D). Similarly to the results based on STAT3 inhibition with C188-9, SESN1&2 KO cells with STAT3 knockdown demonstrated a decreased proliferation rate compared to control cells, indicating that STAT3 plays a critical role in support of cell proliferation in A549 cells (Fig. 5E). To determine if silencing of STAT3 alleviates resistance of SESN1&2 KO cells to cisplatin, we treated control and SESN1&2 KO cells expressing either control or STAT3 shRNA with this drug. While we observed a remarkable difference in the levels of cell death between control and SESN1&2 KO cells expressing shCon, higher levels of cell death were observed in both control and SESN1&2 KO cells expressing shSTAT3. Moreover, the difference in cell death between control and SESN1&2 KO was eliminated, underpinning the important role of STAT3 in Sestrin-regulated cell death following genotoxic stress (Fig. 5F). To evaluate the potential role of MCL1 in the regulation of cell death in control and SESN1&2 KO cells, we silenced MCL1 expression by shRNA-expressing lentivirus and demonstrated that the sensitivity of SESN1&2 KO cells to cisplatin was restored in cells with MCL1 knockdown (Figure S10A,B). Presuming that PTPRD also contributes to cell death and cell proliferation regulation by SESN1&2, we measured the levels of cell death in the SESN1&2 KO cells with ectopic expression of PTPRD. Ectopic expression of PTPRD restored the levels of cell death and proliferation to the levels observed in control SESN1&2-proficient cells, supporting the important mediatory role of PTPRD in the regulation of these processes by SESN1&2 (Figure S11).

## Discussion

Our recent study demonstrated that SESN1 and SESN2 might play an ambiguous role in lung carcinogenesis, supporting tumor growth at an early stage of carcinogenesis but suppressing cell proliferation and facilitating the death of tumor cells at an advanced stage [9]. Therefore, the deficiency of SESN1 or SESN2 supports the growth of tumor xenografts and potentially contributes to cancer progression [7, 9], but their role in carcinogenesis is poorly understood. While Sestrins are established mTORC1 inhibitors, we did not observe any effects of SESN1&2 inactivation on mTORC1 activity under nutrient-rich conditions; however, this does not rule out its potential effects under nutrient or oxygen scarcity [9]. Sestrins are also potential activators of the AKT kinase [37], the major regulator of metabolism and cell viability, however, inactivation of SESN1&2 does not affect phosphorylation of AKT or AKT targets, GSK-β and PRAS40 (Fig. 1A). Therefore, we postulated that some alternative mechanisms might be involved in the control of cell proliferation and cell death by Sestrins in lung adenocarcinoma cells.

To investigate further, we searched for other Sestrin targets that might be responsible for controlling cell proliferation and cell death [38]. Our search was based on a plethora of published data that demonstrated the effect of Sestrins on the regulation of several signaling pathways. For example, the members of the Sestrin family were shown to activate the AMPK-MAPK signaling pathway in T-lymphocytes [26]. The MAPK family members—ERK, JNK, and p38 play important roles in the control of cell division and viability in different cell types and may be targets of Sestrins in lung adenocarcinoma cells. However, we did not observe any difference in the phosphorylation of AMPK and any MAPK proteins in control and SESN1&2 KO cells (Figure S1). SESN2 was recently reported to be an inhibitor of the WNT-β-Catenin signaling pathway, the major regulator of cell stemness and proliferation in colon carcinoma cells [28]. However, we could not detect any effects of SESN1&2 inactivation on the phosphorylation of β-Catenin in lung adenocarcinoma cells (Figure S1). Finally, SESN2 was shown to be a partner of p62 protein and a regulator of its stability and activity [30]. p62 is known for its role as a cargo protein in the delivery of cellular constituents into autophagosomes and, according to recent studies, stimulates hepatocarcinogenesis through control of the viability of preneoplastic cells [39]. We could not demonstrate any difference in p62 levels between control and SESN1&2 KO cells, therefore ruling out the importance of this protein in the control of cell proliferation and cell death by Sestrins in A549 cells (Figure S1).

Analysing expression and phosphorylation of different proteins that potentially contribute to carcinogenesis, we found that phosphorylation of STAT3 was notably increased in single and double SESN1 and/or SESN2 KO cells compared to controls. Surprisingly, no difference in phosphorylation was detected for STAT1, another member of the STAT family, demonstrating that the effects of SESN1&2 inactivation were STAT3-specific (Fig. 1B,C). Interestingly, we could not detect any consequence of Sestrin inactivation on the phosphorylation of the JAK2 kinase, an upstream regulator of STAT3 (Fig. 1C). It indicates that Sestrins regulate STAT3 phosphorylation downstream of JAK2, potentially via regulation of some other STAT3 upstream kinases or phosphatases. In the following studies, we determined that JAK kinases are required for STAT3 phosphorylation in both control and SESN1&2 KO A549 cells (Fig. 2E). Therefore, some phosphatases may be responsible for the regulation of STAT3 phosphorylation by Sestrins. Activation of STAT3 in SESN1&2 KO cells also leads to activation of two crucial STAT3 targets: Cyclin D1 and MCL1, which are critical in supporting cell proliferation and cell viability, respectively. Cyclin D1 promotes cell cycle progression by phosphorylating RB, which releases transcription factors that drive cells into the S phase [40]. Accordingly, RB is hyperphosphorylated in SESN1&2 KO cells, potentially promoting cell cycle progression. In agreement with these data, we have previously demonstrated that ectopic expression of SESN2 leads to downregulation of Cyclin D1 in breast carcinoma MCF7 cells, which in turn leads to suppression of colony growth [8, 25]. Cyclin D1 is overexpressed in many tumors, driving uncontrolled cell proliferation [41]. Similarly, a BCL2 family inhibitor MCL1 is overexpressed in cancers, promoting resistance to anticancer drugs [42, 43]. Elevated MCL1 levels suppress cell death, making cancer cells more resistant to treatments. Targeting MCL1 is a promising strategy for developing new anticancer therapies [43].

To understand how Sestrins regulate STAT3 phosphorylation, we examined several possibilities. These include potential interactions between Sestrins and STAT3 that may suppress STAT3 dephosphorylation by yet-to-be-identified phosphatase(s), negative regulation of cytokine secretion involved in STAT3 activation, or suppression of ROS production by Sestrins taking into consideration the well-established antioxidant activity of Sestrins [6, 30, 32, 44]. Many protein tyrosine phosphatases are redox-dependent proteins that may be inactivated by the excess ROS produced in SESN1&2-null cells [45, 46]. We could not find evidence supporting the impact of any of these potential mechanisms in the control of STAT3 phosphorylation by Sestrins (Fig. 2A-D, S2). We also ruled out any potential impact of GATOR2 in STAT3 regulation by Sestrins as knockdown of a critical GATOR2 compound – MIOS did not affect STAT3 phosphorylation (Fig. 2B). While the regulation of STAT3 phosphorylation is yet to be understood, we demonstrated that it is dependent on the JAK activation but not on EGFR (Fig. 2E-F); the former is often overactivated in lung cancer cells [47]. Therefore, searching for the mechanism of regulation of STAT3 by Sestrins, we compared gene expression in control and SESN1&2 KO cells to find potential regulators of STAT3 activity. According to our analysis, transcripts of *PTPRD*, *H19*, *HACD4/PTPLAD2*, *PTPRB*, and *BPIFA1* genes were downregulated in SESN1&2 KO cells (Fig. 3A-B). The most prominent candidate on the role in the regulation of STAT3 phosphorylation by Sestrins that appeared in our search was the protein tyrosine phosphatase receptor delta (PTPRD), whose inhibitory effect on STAT3 phosphorylation in different cancer types was demonstrated in several studies [33, 48]. PTPRD is inactivated in many human cancers and potentially plays a role as a tumor suppressor through STAT3 inhibition [49]. PTPRD also provides sensitivity to radiotherapy in nasopharyngeal carcinoma cells [50]. It was also demonstrated that PTPRD could regulate PD-L1 via its effects on STAT3 in human hepatocellular carcinoma, and consequently, enhanced STAT3 activity may support tumor immune evasion [51]. PTPRB, another member of the PTPR family, may also inhibit STAT3, however, its role in the dephosphorylation of STAT3 has not yet been proven. Non-coding H19 RNA is another STAT1&2 target that might be involved in STAT3 regulation. As demonstrated in endothelial cells, knockdown of H19 leads to STAT3 phosphorylation, and therefore, downregulation of H19 in response to SESN1&2 inactivation might cause STAT3 dephosphorylation [52]. Protein tyrosine phosphatase-like A domain-containing 2 (HACD4/PTPLAD2) is a potential tumor suppressor in squamous carcinoma responsible for the inhibition of STAT3 phosphorylation [53]. Lipid-binding antimicrobial Bactericidal/permeability-increasing fold containing family A (BPIFA1) is a negative regulator of pro-inflammatory response in the lung and might inhibit STAT3 through the downregulation of IL6, the potent activator of the JAK2-STAT3 pathway [54]. Focusing on PTPRD, we demonstrated that ectopic expression of PTPRD decreases STAT3 phosphorylation in A549 SESN1&2 KO cells (Fig. 3F-G). Therefore, PTPRD may play a major role in the STAT3 regulation by Sestrins. However, potential cooperation among the Sestrin targets identified in our study cannot be excluded. We have demonstrated that downregulation of the PTRPD in SESN1&2 KO cells is not caused by accelerated degradation of its mRNA (Fig. 3E). Therefore, Sestrins should be responsible for the regulation of PTPRD transcription. However, the mechanism of transcriptional regulation by SESN1&2 is obscure, as most Sestrin activities occur in the cytoplasm or on the mitochondria [14, 32, 55]. It was reported that SESN2 may regulate the activity of the NRF2 transcription factor by supporting KEAP1 degradation via specific autophagy, which leads to the release of NRF2, followed by transcriptional activation of many genes involved in the antioxidant response [30]. Alternatively, SESN2 might directly support NRF2 transcriptional activity [56]. A549 cells bear mutations in the KEAP1 gene [57], and the regulation of NRF2 by means of KEAP1 degradation in these cells is unlikely. Sestrins may regulate gene expression by modulating the transcriptional activity of NRF2 or other factors to be identified. Additionally, we detected a portion of SESN2 protein in the nucleus, suggesting that SESN2 may have a currently unknown role in transcriptional regulation (data not shown).

Targeting STAT3 in combination with chemotherapeutic or immunotherapeutic drugs, may improve cancer treatment outcomes by suppressing cell proliferation and sensitising cancer cells to death [58, 59]. Being p53-activated genes and regulators of cell viability [8], SESN1&2 may contribute to chemotheraphy-induced apoptosis in cancer cells. Therefore, we examined the role of Sestrins in DNA damage response and the potential crosstalk of Sestrins with the STAT3 pathway. Our findings show that cisplatin treatment significantly upregulates SESN1 and SESN2 expression in A549 cells via p53 activation (Fig. 4E) [8]. Furthermore, we demonstrated that SESN1&2 strongly contribute to cisplatin-induced cell death in A549 cells as SESN1&2-null cells have an increased apoptotic resistance and form substantially more colonies in a long-term colony-forming assay (Fig. 4A,C). Tumor suppressor p53, activates SESN1 and SESN2 transcription and is critical for cell death in response to genotoxic stress. Sestrins’ status does not affect cisplatin-induced cell death in the absence of p53 (Fig. 4B). Being direct targets of p53, Sestrins could contribute to the control of the DDR. However, SESN1&2 do not significantly impact the expression and phosphorylation of DDR regulators or p53 activity, as seen by p53 phosphorylation and DR4 upregulation (Fig. 4E). Altogether, these data suggest that Sestrins’ role in supporting p53-induced cell death may be downstream of p53. Sestrins’ effect on cell death is not limited to cisplatin and is also observed with the topoisomerase inhibitor etoposide (Fig. 4C) [8].

Presumably, Sestrins contribute to p53-dependent mechanisms by modulating some collateral signaling pathways involved in the control of proliferation and cell death – processes that are both closely associated with STAT3. To examine this potential connection, we demonstrated that inhibiting STAT3 in SESN1&2 KO cells considerably reduced cell proliferation and restored cisplatin-induced cell death to levels observed in control cells (Fig. 5). Similar restoration of cell death was observed when cells were treated with ruxolitinib, a JAK kinase inhibitor, indicating the importance of the JAKs on STAT3 activity (Figure S8). Silencing of MCL1 or ectopic expression of PTPRD also restored cell death in SESN1&2 KO cells (Figure S10,S11). Nevertheless, silencing of MIOS did not affect either STAT3 phosphorylation or cisplatin-induced cell death (Fig. 2B,S7), supporting our conclusion that Sestrin regulation of STAT3 is GATOR2-independent. Therefore, restoration of Sestrin activity or STAT3 inhibition in SESN1&2-deficient cancer cells may be a potential strategy to improve the treatment outcome for patients with cancers bearing mutations in *SESN1* or *SESN2* genes.

STAT3 is upregulated and activated in many human cancers, including NSCLC, prostate cancers, and melanomas [21, 23]. STAT3 promotes carcinogenesis by inhibiting cell death, supporting angiogenesis and cell stemness, providing resistance to chemotherapies, and facilitating immune evasion through inflammatory signaling [20, 22, 60–62]. Here, we described a new mechanism of STAT3 activation in cancers mediated by the downregulation of p53-inducible *SESN1* and *SESN2* genes. While we demonstrated the importance of STAT3 in acceleration of cell proliferation and inhibition of cell death by SESN1&2, the impact of these proteins in the regulation of other STAT3-regulated processes involved in carcinogenesis will be determined in future studies with the help of in vivo models. Future studies should explore the crosstalk between STAT3 and mTORC1 pathways in more detail. While SESN1&2 inactivation does not affect mTORC1 in nutrient-rich conditions, Sestrins may inhibit mTORC1 under nutrient or oxygen scarcity, common in tumors. mTORC1 is a key regulator of cell proliferation and death [13], and understanding its potential inhibition by Sestrins could add complexity to carcinogenesis.

Besides NSCLC, STAT3 activation is often observed in non-Hodgkin lymphomas, including follicular lymphomas (FL). Our studies demonstrated that FL are often characterised by the inactivation of SESN1 [63]. STAT3 inhibition imparts sensitivity of lymphoma cells to chemotherapeutic drugs [64], and activation of Sestrins might be a potential mechanism to improve the outcome of anticancer therapies. Likewise, SESN2 is found downregulated in colon cancer [65], where STAT3 is often hyperactivated and contributes to carcinogenesis [66]. As SESN1 or SESN2 expression is often downregulated in different tumors due to inactivation of p53 or chromatin modifications, activation of SESN1&2 expression via alternative mechanisms such as treatment with metformin or other inhibitors of metabolic pathways [18] may restore Sestrin function and intensify the therapeutic effects of the conventional as well as emerging anticancer therapies. Furthermore, Sestrins can also be potentially activated by small molecules that can interact with the leucine-binding pocket of the Sestrins, enhancing their activity [67]. Additionally, activation of Sestrins in healthy people may be used to slow down ageing and prevent development of cancer and other age-related diseases.

## Materials and methods

### Cell culture and treatment

A549 cells were obtained from American Type Culture Collection (ATCC) and cultured in DMEM containing 4.5 g/L glucose, supplemented with 2 mM L-glutamine, 5% penicillin/streptomycin (v/v), and 10% fetal bovine serum (v/v). To induce DNA-damage and cell death, cells were treated with 20 µM cisplatin for 24–48 h. To analyse the impact of different signaling pathways in cell death, cells were treated with N-acetylcysteine (0.1–5 mM) for 4 h, ruxotinib (0.5 µM) for 6 h, or erlotinib (0.5 µM) for 6 h. To inhibit transcription, cells were treated with Actinomycin D (10 µg/ml). To inhibit STAT3, cells were treated with C188-9 (Sigma-Aldrich).

### Constructs, cells, and lentiviral infection

The A549 SESN1 and SESN2 KO cells, expressing sgSESN1-1, sgSESN1-2, sgSESN2-1, sgSESN2-2, sgSESN1&2–1, and sgSESN1&2–2 cells were previously described [9]. The shCon(shLuc), shSESN1 (shSESN1-2), shSESN2 (shSESN2-1), and shMIOS, Flag-SESN1, HA-SESN2 constructs were previously described [9, 14, 19, 25]. The sequence targeting STAT3 was 5’-GCAGCAGCTGAACAACATG-3’. The sequence targeting MCL1 was 5’-GCAGCAGCTGAACAACATG-3’. To generate SESN1&2-silenced cells, the cells were infected with indicated lentiviral constructs and selected in the medium with puromycin (400 µg/ml) and hygromycin (400 µg/ml) described previously [9, 18]. To ectopically express SESN1 and SESN2, cells were infected with the SESN2-expressing lentiviral vector as previously described [14]. To ectopically express PTPRD, cells were infected with the PTPRD-expressing chicken lentiviral vector. The plasmid pCDF1-PTPRD-WT was a gift from Todd Waldman (Addgene plasmid # 25,642; http://n2t.net/addgene:25642; RRID:Addgene_25642) [68].

### Cell lysis, immunoprecipitation, and immunoblot analysis

For IP and IB analyses, cells were lysed in NP40 or RIPA buffer, respectively, and analysed as described previously [14]. Cells were placed on ice, washed twice with ice-cold PBS, and lysed in a corresponding buffer containing protease and phosphatase inhibitors (Roche). Protein concentration was estimated with Pierce BCA Protein Assay Kit (Thermo scientific), and 30 µg of protein was used for each load. The antibodies used: rabbit phospho-STAT3 (Tyr705) (D3A7) #9145, mouse STAT3 (124H6) #9139, rabbit phosphor-STAT1 (Tyr701) (58D6) #9167, rabbit STAT1 (91K9Y) #14994, rabbit phosphor-JAK2 (Tyr1007/1008) #3771, rabbit JAK2 (D2E12) #3230, rabbit phospho-GSKβ (Ser9) #5558, rabbit GSK3β (27C10) #9315, rabbit phospho-PRAS40 (Thr246) #2997, rabbit PRAS40 (D23C7) #2691, rabbit PRAS40 (D23C7) 2691, rabbit phospho-p70S6K (T389) (108D2) #9234 T, rabbit p70S6K #9202, rabbit phospho-4E-BP1 (Ser65) #9451 T, rabbit-4E-BP1 #9452, rabbit phospho-S6 Ribosomal Protein (Ser235/236) #2211, rabbit S6 Ribosomal Protein (5G10) #2217, rabbit phospho-ULK1 (Ser757) #6888, rabbit ULK1 (D8H5) #8054, rabbit phospho-AKT (Ser473) (D9E) #4060, rabbit AKT (C67E7) #4691, rabbit phospho-ERK (D13.14.4E) #4370, rabbit ERK (137F5) #4695, rabbit phospho-JNK (Thr183/Tyr185) (81E11) #4668, rabbit phospho-p38 MAPK (Thr180/Tyr182) (93F9) #4511, rabbit p38 MAPK (D13E1) #8690, rabbit phospho-AMPK (40H9) #2535, rabbit AMPK #2532, rabbit MCL-1 (D35A5) #5453, rabbit Cyclin D1 (92G2) #2978, rabbit phospho-RB (Ser780) #3590, mouse Rb (4H1) #9309, rabbit phospho-β-Catenin (Ser552) #5651, rabbit β-Catenin (6B3) #9582, mouse p62 (D5L7G) #88588, rabbit MIOS (D12C6) #13557, rabbit phospho-Histone H2AX (Ser139) (20E3) #9718, rabbit H2AX #2595, mouse phospho-p53 (Ser15) (16G8) #9286, rabbit phospho-CHK1 (Ser345) (133D3) #2348, mouse CHK1 (2G1D5) #2360, rabbit phospho-CHK2 (Thr68) (C13C1) #2197, rabbit CHK2 #2662, rabbit phospho-BRCA1 (Ser1524) #9009, rabbit BRCA1 #9010, rabbit DR4 (D9S1R) #42533, rabbit phosphor-EGFR (Tyr1068) (D7A5), rabbit EGFR (D38B1) #4267 were from Cell Signaling Technology. Mouse p53 (DO-7) (sc-47698), GFP (C-2) (sc-390394) and β-Actin (C4) (sc-47778) were from Santa Cruz Biotechnology. Rabbit SESN2 antibody (10795–1-AP) was from Proteintech Inc [14] and was used for the WB analyses. Mouse SESN2 antibody (41-K) (sc-101249) was used for IP analyses. Rabbit anti-SESN1 antibody was previously described [37, 63].

### Analysis of gene expression by RNA-seq, RNA library preparation, and sequencing

Total RNA was isolated from control and SESN1&2 KO A549 cells (in triplicates), using the MagNA Pure Compact RNA Isolation Kit (Roche, Switzerland) on a MagNA Pure Compact Instrument (Roche) according to the manufacturer’s instructions. Isolated RNA was quantified with a Qubit 2.0 Fluorometer (Thermo Fisher Scientific, USA); the quality of samples was assessed on an Agilent 2100 Bioanalyzer (Agilent Technologies, USA) with the RNA Integrity Number (RIN) measurement. Samples with RIN > 7 were only used for library preparation. Transcriptome libraries were prepared using the TruSeq Stranded mRNA Library Prep Kit (Illumina, USA) following the manufacturer’s protocol. cDNA quality and concentration were assessed on Qubit 2.0 Fluorometer and Agilent 2100 Bioanalyzer, respectively. Cluster densities were optimised by quantitative PCR using a Rotor-Gene Q 5 plex HRM instrument (Qiagen, Netherlands). Obtained cDNA libraries were sequenced on a NextSeq 500 System (Illumina) under the 76 bp single-end mode.

The quality control of raw RNA-seq data was carried out using FastQC 0.11.5. Trimming and filtering of reads, removal of residual adapters was done with Trimmomatic 0.39 [69]. The ratio of rRNA reads (the efficacy of PolyA extraction) was assessed via mapping the reads to the complete human rRNA sequences using Bowtie2 2.3.5.1 [70]. Then, STAR 2.7.9a was used for splice-aware mapping reads to the reference hg38 human genome supplied with GTF (Ensemble annotation, release 108) [71]. Next, read counts per gene were quantified using featureCounts tool from the Subread 1.6.0 package [72]. Also, we performed 3’-bias analysis with geneBody_coverage.py script from RSeQC 2.6.4 toolkit in order to ensure the absence of 3’-bias differences between the samples. The differential expression analysis was performed using edgeR 4.0.2 package (R 4.3.2, Bioconductor) [73]. Gene Ontology (GO) enrichment (overrepresentation) analysis was performed using topGO 2.54.0 package [74] independently for top-50, 100, 250, etc. up- or down-regulated genes. We also performed transcription factor target enrichment analysis using ChEA3 web service [75].

### Analysis of relative gene expression by qPCR

Following RNA isolation by TRIzol reagent, cDNA was synthesised by following the Invitrogen SuperScript™ III Reverse Transcriptase instructions. No-RT controls were prepared in parallel without the addition of SuperScript III RT enzyme. The cDNA product was aliquoted and frozen or used immediately for qPCR analysis. The cDNA templates were mixed with Power SYBR® Green PCR Master Mix according to manufacturer instructions. Comparative qPCR analysis was performed using the Mx3000P Agilent qPCR system. As our primers were designed to give 200 bp amplicons, annealing and extension times were limited to 30 s to minimise non-specific amplification. Following PCR, no-RT controls were examined to have no amplification. To prevent non-specific amplification on genomic DNA, primers were designed to span exon junctions. The amplicons were also inspected on 1% agarose gel, and no-RT controls were expected to show no bands or a faint non-specific band at a different molecular weight than the experimental band, for example, primer dimer < 100 bp. Primers were designed with aid of Primer-BLAST [76].

The primers used are as follows:

- β-actin, 5’-GAGCACAGAGCCTCGCCTTT-3’ and 5’-GATGCCTCTCTTGCTCTGGG-3’
- H19, 5’-ACGTGACAAGCAGGACATGA-3’ and 5’-TGAGCTGGGTAGCACCAT-3’
- PTPRD, 5’-GTGGGGTCACCAATGCCTTA-3’ and 5’-TGCTCTCGGTCACTACAGGA-3’
- PTPRB, 5’-GCTCAACATCACTGTGGGAA-3’ and 5’-ATGTTTGACACGAAGCTGGA-3’
- HACD4/PTPLAD2, 5’-GTTCTGTGGCCACTCTTGGA-3’ and 5’-CGGATTGGCAAAGTCGCATC-3’
- BPIFA1, 5’-TGAAGCCTGGAGGAGGTACT-3’ and 5’-GAGCTTTATGCCGAGAGGGA-3’

### Colony-forming assay

Five hundred cells were plated on a well of 6-well plates. 24 h later cells were incubated with cisplatin (20 µM) or etoposide (20 µM) for 6 h, and then the medium was replaced with fresh medium. 2 weeks later cells were fixed with 100% methanol and stained with methylene blue as previously described [8].

### Analysis of cell death

Cells were treated with a drug for 24 h, harvested and washed in PBS. Pellets were re-suspended in AnnexinV binding buffer and incubated with anti-AnnexinV-FITC antibody and propidium iodide (PI) following the manufacturer’s instructions (BD Biosciences). Cells were analysed on the BD Accuri C6 instrument and data were analysed using BD Accuri C6 Software.

### Analysis of cell proliferation/ growth curves

For analysis of cell proliferation, cells were plated onto 6 cm culture dishes at 50,000 cells/dish and counted every 24 h for 5 days. 50,000 cells were plated onto 6 cm dishes. On the following day (Day 0), the cells were counted and treated with varying concentrations of the drug or left untreated. Day 1 marks the first 24 h after treatment with a drug. Cells were counted using a Neubauer Improved Haemocytometer. Four outer corner squares on the haemocytometer were counted. The obtained count was divided by four to obtain a representative average. A minimum of six averages collected per condition. Three dishes per condition were counted to obtain three biological repeats (*n* = 3). Counts were converted to number of cells × 10^6. The counts were performed every 24 h for five days.

### Statistical analysis

Statistical analysis was performed with GraphPad Prism software version 9.0 (GraphPad Software, La Jolla, CA) using two-tailed Student’s t-test, one-way, or two-way ANOVA followed by Tukey’s test for multiple comparisons. Statistical significance was defined as *p* < 0.05. Results are presented as mean ± standard deviations or standard errors as indicated in the legends.

## Supplementary Information

Supplementary Material 1.

## Data Availability

No datasets were generated or analysed during the current study.

## Abbreviations

mTORC1/2: Mammalian target of rapamycin complex ½
ROS: Reactive oxygen species
NSCLC: Non-small cell lung cancer
STAT: Signal transducer and activator of transcription
JAK: Janus kinase
