# Supplementary material for: p53-regulated SESN1 and SESN2 regulate cell proliferation and cell death through control of STAT3
Source: Cell Commun Signal. 2025 Feb 22;23:105. doi: 10.1186/s12964-025-02104-3 (PMC11846189; doi:10.1186/s12964-025-02104-3)
Supplement: Supplementary file 1 — Supplementary Material 1. [file 12964_2025_2104_MOESM1_ESM.docx]

**Supplemental data**

**p53-regulated SESN1 and SESN2 regulate cell proliferation and cell death through control of STAT3**

Alexander Haidurov, Andrei O. Zheltukhin, Anastasiya V. Snezhkina, George S. Krasnov, Anna V. Kudryavtseva, and Andrei V. Budanov

A B C


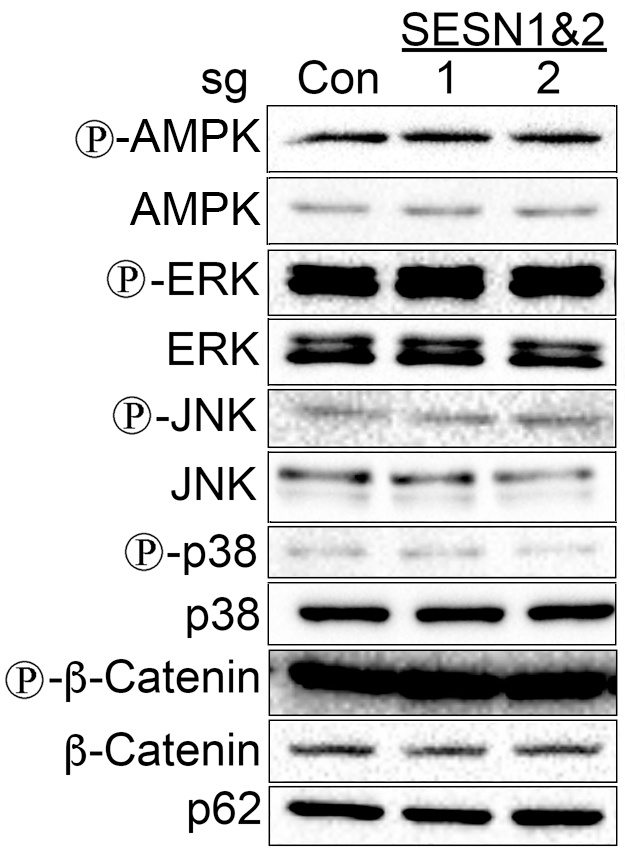

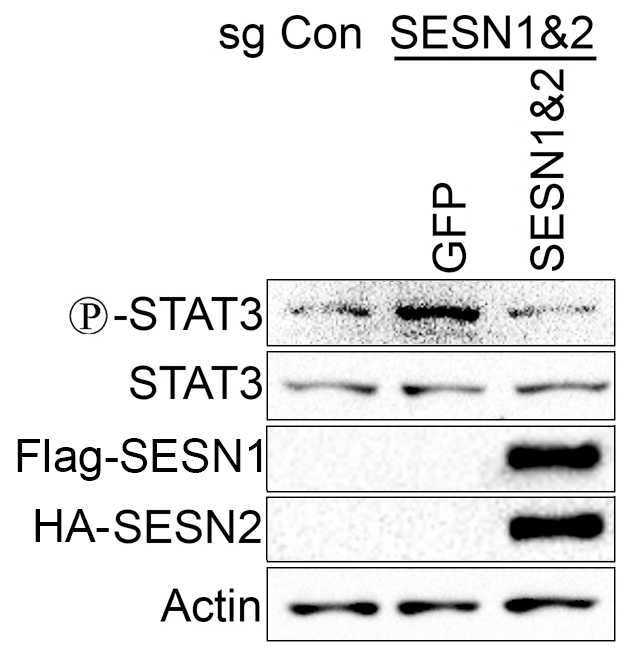

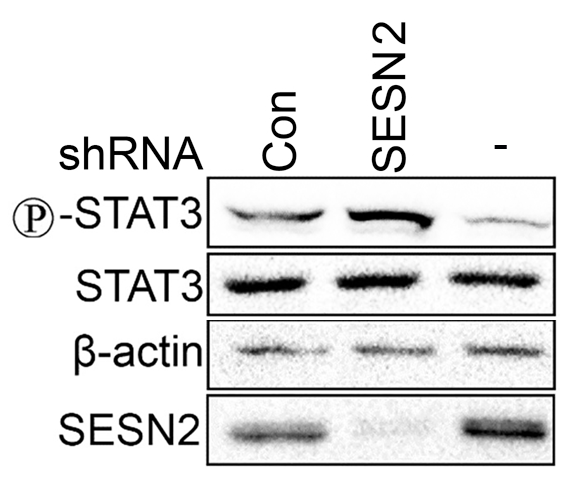


**Figure S1. SESN1&2 suppress STAT3 phosphorylation but dispensable for control of other signaling pathways.** A) Immunoblot analysis of phosphorylation and expression of the components of several signaling pathways controlling cell growth and proliferation in control (sgCon) and SESN1&2 KO (sgSESN1&2) cells. B) Ectopic expression of both SESN1&2 but not GFP protein restores low STAT3 phosphorylation in SESN1&2 KO cells. C) SESN2 silencing in H460 cells leads to upregulation of STAT3 phosphorylation. For immunoblot analysis in A were used the same lysates as in Figure 1A and B. The expression of Flag-SESN1, HA-SESN2 and shRNA SESN2 was achieved by infection with corresponding recombinant lentiviruses.


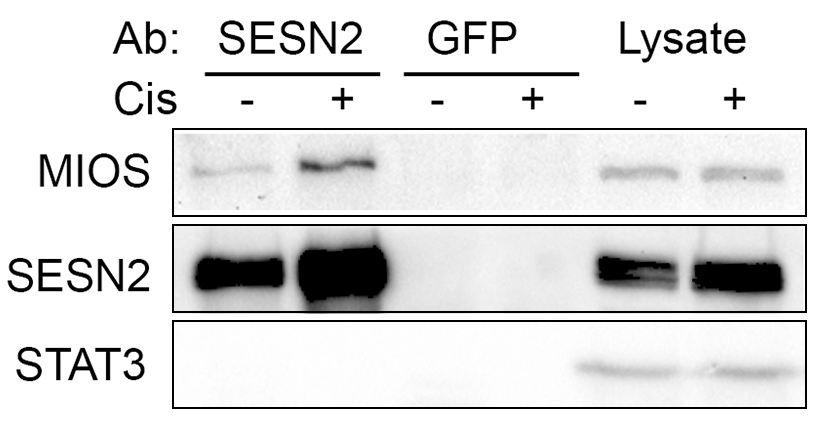


**Figure S2. SESN2 does not interact with STAT3.** SESN2 was immunoprecipitated using an anti-SESN2 antibody. Alternatively, an anti-GFP antibody was used for immunoprecipitation to evaluate potential non-specific interactions. The indicated proteins were then detected by immunoblotting with their corresponding antibodies, enabling the visualization and analysis of specific protein interactions.


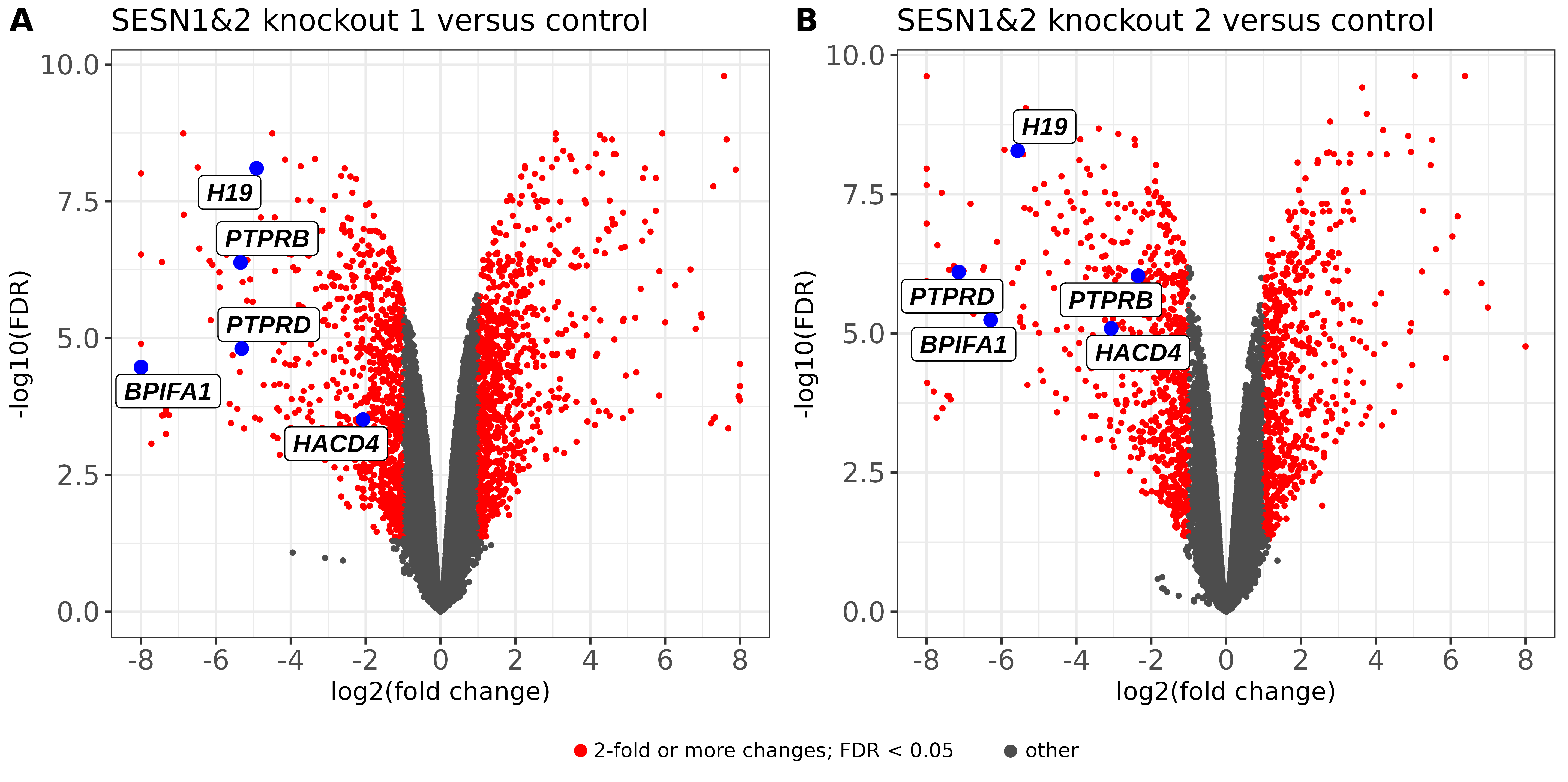


**Figure S3. Volcano plot of RNAseq analysis of difference in gene expression demonstrating expression of potential regulators of STAT3.** RNAseq was used to analyse relative gene expression in Control, SESN1&2 KO1 (A) and SESN1&2 KO2 (B) cells, data were plotted and potential regulators of STAT3 were indicated by blue dots.

Volcano plot showing the amplitude (log2 of fold change) and statistical significance (FDR) of gene expression changes in the SESN1&2 KO1 (A) and SESN1&2 KO2 (B) variants of Sestrins knockouts compared to the controls. Genes with 2-fold or more fold expression changes that passed the FDR < 0.05 threshold are marked in red. Genes of interest of this paper are marked in blue.

**A B**

| TF | Target genes (among top-100 upreg.) | FET p-value | Odds ratio |
| --- | --- | --- | --- |
| CTCF | 16 | 0.02836 | 1.792 |
| STAT1 | 9 | 0.03028 | 2.18 |
| STAT2 | 4 | 0.03747 | 3.309 |
| STAT3 | 15 | 0.03916 | 1.744 |
| REST | 5 | 0.06636 | 2.391 |
| CBX2 | 14 | 0.06861 | 1.626 |
| JUND | 4 | 0.07223 | 2.639 |
| FOSL1 | 4 | 0.07484 | 2.606 |
| HNF4A | 17 | 0.09925 | 1.466 |
| TCF7L2 | 5 | 0.1017 | 2.089 |
| MAFK | 12 | 0.1139 | 1.535 |
| ZNF263 | 13 | 0.1307 | 1.468 |
| ESR1 | 5 | 0.1414 | 1.869 |
| RFX5 | 4 | 0.1687 | 1.904 |
| JUN | 4 | 0.1849 | 1.832 |
| SREBF1 | 4 | 0.1878 | 1.82 |
| FOS | 6 | 0.1906 | 1.591 |
| TBP | 12 | 0.2026 | 1.353 |
| MAX | 12 | 0.2059 | 1.348 |
| SPI1 | 7 | 0.2164 | 1.469 |

| TF | Target genes (among top-200 upreg.) | FET p-value | Odds ratio |
| --- | --- | --- | --- |
| JUND | 10 | 0.001412 | 3.33 |
| CBX2 | 32 | 0.001737 | 1.858 |
| ESR1 | 13 | 0.004355 | 2.441 |
| FOSL1 | 9 | 0.005078 | 2.949 |
| HNF4A | 37 | 0.008739 | 1.593 |
| CTCF | 44 | 0.01702 | 1.466 |
| ESRRA | 4 | 0.02248 | 3.906 |
| PRDM1 | 23 | 0.02879 | 1.596 |
| NFIC | 11 | 0.03092 | 1.977 |
| TCF12 | 13 | 0.03752 | 1.806 |
| STAT1 | 14 | 0.0499 | 1.689 |
| MYOD1 | 14 | 0.05142 | 1.681 |
| NR3C1 | 5 | 0.05259 | 2.552 |
| FOSL2 | 8 | 0.05434 | 2.011 |
| STAT3 | 25 | 0.05842 | 1.447 |
| FOXP2 | 4 | 0.06412 | 2.745 |
| TEAD4 | 8 | 0.06737 | 1.916 |
| JUN | 8 | 0.08215 | 1.83 |
| SREBF1 | 8 | 0.08452 | 1.818 |
| FOS | 12 | 0.0909 | 1.587 |

**Table S4.** The results of transcription factor targets enrichment (ChEA3) for top-100 (A) and top-200 (B) genes upregulated after SESN1&2 KO. STAT family transcription factors are highlighted in colour. FET - Fisher's exact test.

**A**

| TF | Target genes (among top-100 downreg.) | FET p-value | Odds ratio |
| --- | --- | --- | --- |
| CEBPB | 6 | 0.003206 | 4.451 |
| STAT3 | 16 | 0.02482 | 1.823 |
| NR3C1 | 15 | 0.0277 | 1.832 |
| NFIC | 7 | 0.03207 | 2.426 |
| MYOD1 | 9 | 0.03442 | 2.126 |
| TCF12 | 10 | 0.03582 | 2.022 |
| TFAP2A | 15 | 0.04998 | 1.68 |
| ZNF217 | 12 | 0.06471 | 1.71 |
| REST | 9 | 0.06647 | 1.859 |
| FOSL1 | 4 | 0.07915 | 2.553 |
| STAT1 | 4 | 0.08054 | 2.537 |
| STAT5A | 3 | 0.08074 | 3.061 |
| MAFK | 14 | 0.08179 | 1.577 |
| GATA3 | 9 | 0.09192 | 1.731 |
| TCF3 | 13 | 0.1015 | 1.542 |
| CTCF | 13 | 0.1092 | 1.52 |
| STAT2 | 3 | 0.1344 | 2.422 |
| NR2F2 | 5 | 0.1475 | 1.841 |
| GATA2 | 7 | 0.1533 | 1.632 |
| JUND | 10 | 0.1542 | 1.493 |

**B**

| TF | Target genes (among top-200 downreg.) | FET p-value | Odds ratio |
| --- | --- | --- | --- |
| NFIC | 16 | 4.69E-04 | 2.788 |
| NR3C1 | 32 | 8.17E-04 | 1.955 |
| MYOD1 | 19 | 0.001781 | 2.248 |
| TCF12 | 17 | 0.002097 | 2.327 |
| STAT2 | 8 | 0.004502 | 3.262 |
| STAT3 | 30 | 0.006912 | 1.706 |
| GATA3 | 20 | 0.007103 | 1.925 |
| TFAP2A | 30 | 0.00851 | 1.677 |
| ESR1 | 12 | 0.01227 | 2.203 |
| STAT1 | 8 | 0.01741 | 2.543 |
| CEBPB | 18 | 0.03122 | 1.682 |
| REST | 9 | 0.03414 | 2.107 |
| CTCF | 26 | 0.03552 | 1.517 |
| JUND | 21 | 0.04074 | 1.565 |
| ZNF217 | 21 | 0.06019 | 1.49 |
| CBX2 | 25 | 0.06946 | 1.417 |
| MAFK | 25 | 0.07547 | 1.402 |
| GATA2 | 25 | 0.07896 | 1.394 |
| FOSL1 | 6 | 0.1048 | 1.907 |
| TCF3 | 23 | 0.1066 | 1.358 |

**Table S5.** The results of transcription factor targets enrichment (ChEA3) for top-100 (A) and top-200 (B) genes upregulated after SESN1&2 KO. STAT family transcription factors are highlighted in colour. FET - Fisher's exact test.


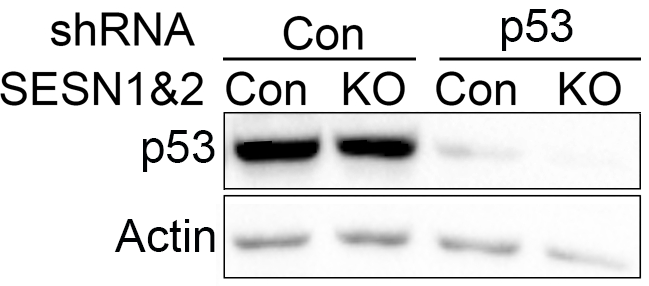


**Figure S6. Knockdown of p53 in control and SESN1&2 KO cells treated with cisplatin.** p53 was silenced by shRNA-expressing lentivirus in control and SESN1&2 KO A549 cells, cells were treated with cisplatin (20 µM) for 24 h, and expression of the indicated proteins was analysed by immunoblot.


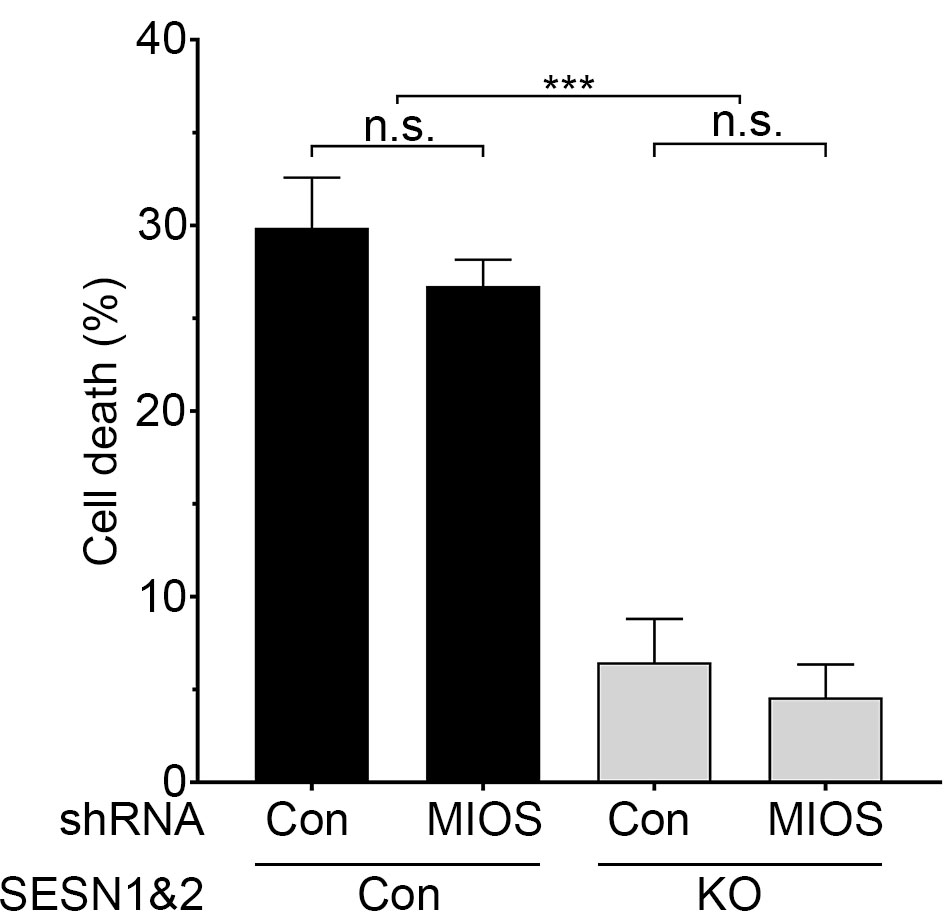


**Figure S7. Sestrin regulate cell death in response to DNA-damage in a GATOR2-independent manner.** Control and SESN1&2 KO cells expressing either control or MIOS shRNA were treated with cisplatin (20 µM) for 24 h and the levels of cell death were analysed by annexin V staining followed by flow cytometry. The data are presented as mean ±S.D. (n = 3). P values were calculated using two-way ANOVA, followed by Tukey’s post-test comparison. n.s. - non- specific; p***≤0.001.


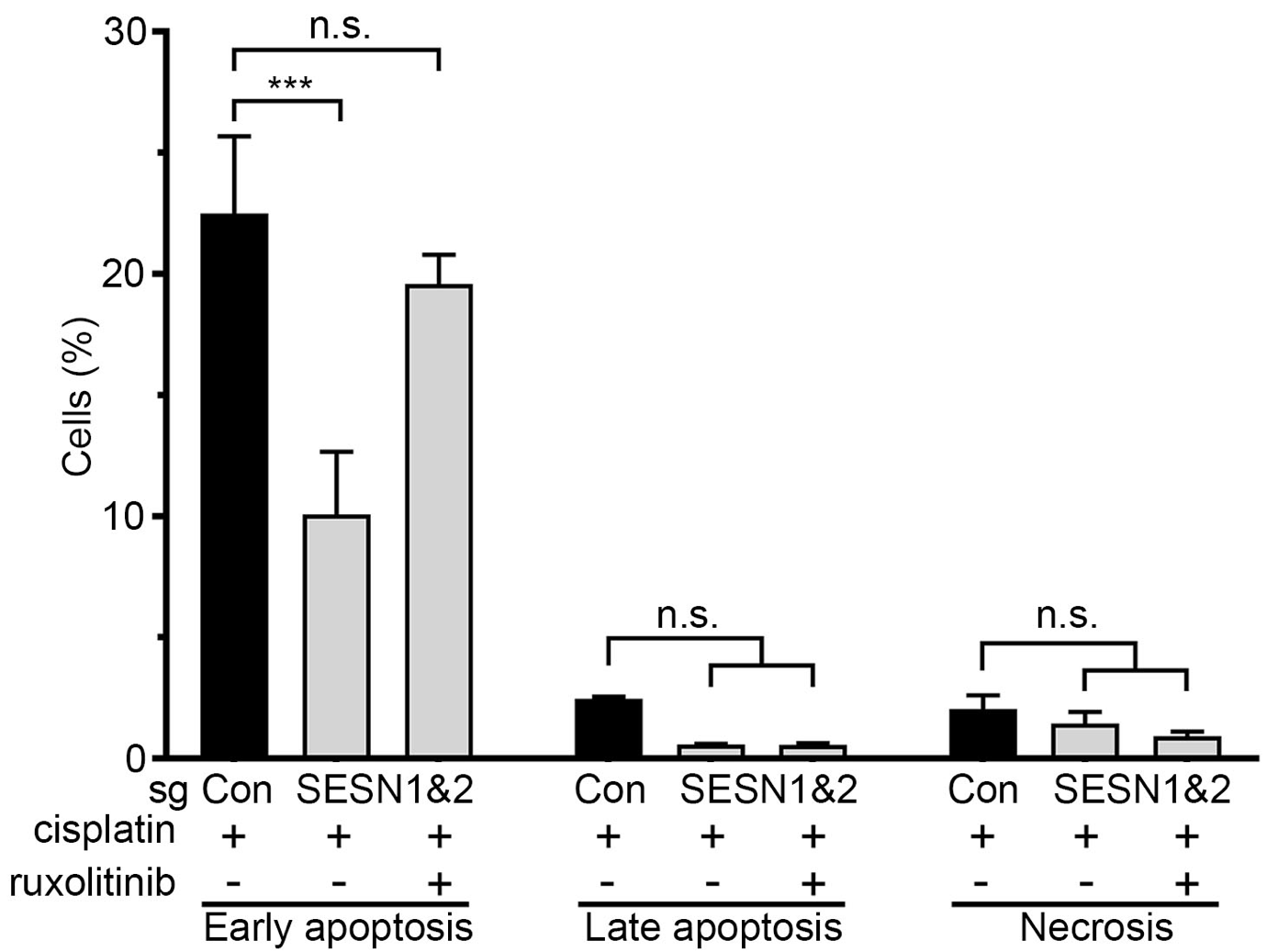


**Figure S8. Treatment with ruxotinib restores the levels of cell death in SESN1&2 KO cells to the levels observed in control cells.** Control and SESN1&2 KO cells were treated for 24 h with cisplatin (20 µM) in the presence of different concentrations of ruxotinib and the levels of cell death were determined by Annexin;PI staining followed by flow cytometry. The data are presented as mean ±S.D. (n = 3). P values were calculated using two-way ANOVA, followed by Tukey’s post-test comparison. n.s. - non- specific; p***≤0.001.


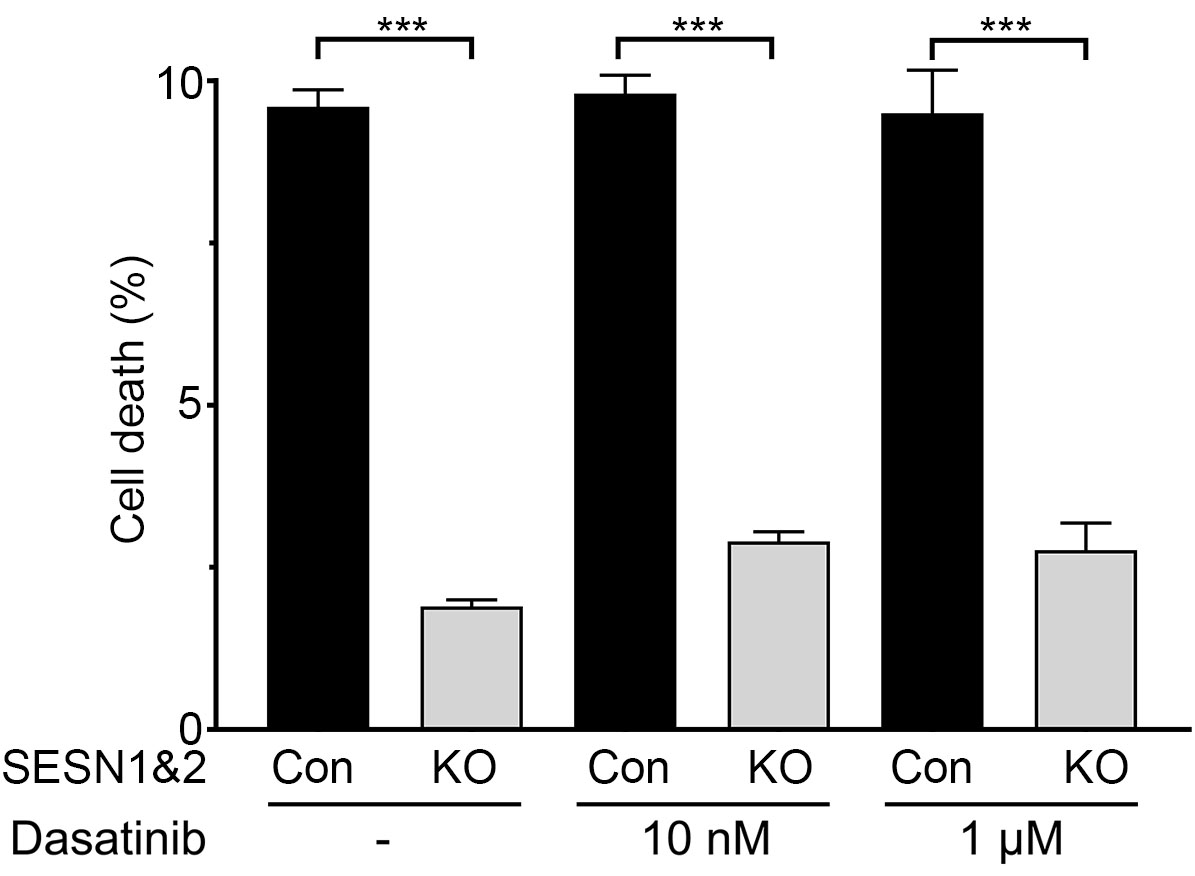


**Figure S9. Treatment with dasatinib has no effect on the cell death induced by cisplatin.** Control and SESN1&2 KO cells were treated with cisplatin (20 µM) in the presence of different concentrations of dasatinib and the levels of cell death were determined by Annexin V staining followed by flow cytometry. The data are presented as mean ±S.D. (n = 3). P values were calculated using two-way ANOVA, followed by Tukey’s post-test comparison. n.s. - non- specific; p***≤0.001.

A B


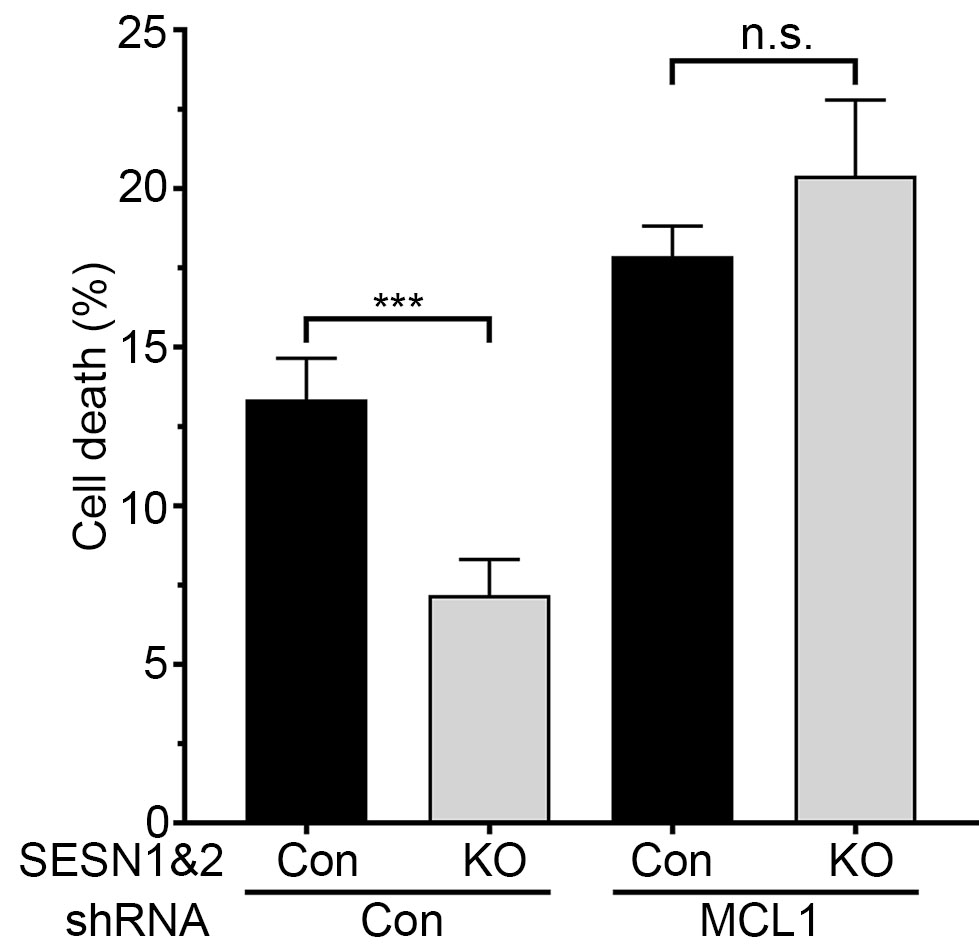

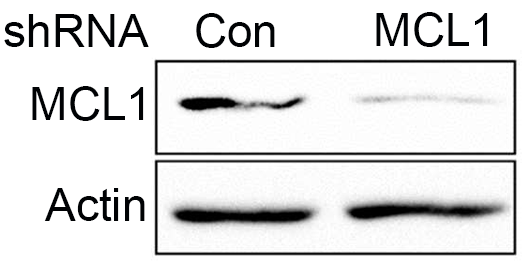


**Figure S10. Knockdown of MCL1 reverses cell death levels in SESN1&2 KO cells.** MCL1 was silenced by shRNA-expressing lentivirus in control and SESN1&2 KO cells. A) Indicated cells were treated with cisplatin (20 µM) for 24 h and the levels of cell death were analysed by Annexin V staining followed by flow cytometry. B) Analysis of MCL1 knockdown in SESN1&2 KO cells by immunoblot.


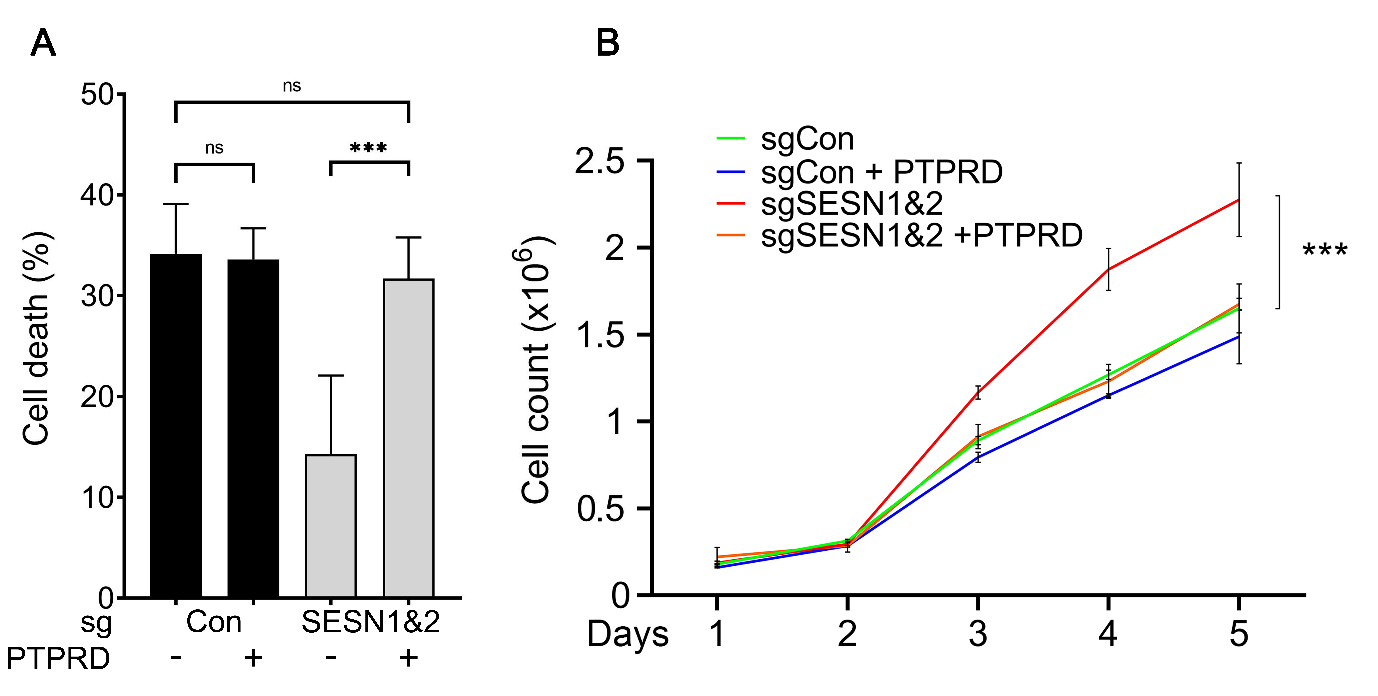


**Figure S11. Ectopic PTPRD expression reverses the effects of SESN1&2 inactivation on cell death and proliferation.** The PTPRD-expressing construct was delivered in a lentiviral vector. (A) Control and SESN1&2 KO cells with and without PTPRD were treated with cisplatin for 24 h and the levels of cell death were defined by Annexin V staining followed by flow cytometry. (B) 50000 cells were plated on 6-cm culture dishes and the number of cells of indicated genotype was calculated every 24 h during 5 days by haemocytometer.
